# Alternative Splicing of NAC Transcription Factor Gene *CmNST1* Is Associated with *Naked Seed* Mutation in Pumpkin, *Cucurbita moschata*

**DOI:** 10.3390/genes14050962

**Published:** 2023-04-23

**Authors:** Qiong Shen, Yiqun Weng

**Affiliations:** 1 College of Horticulture, Shanxi Agricultural University, Taiyuan 030031, China; 2 USDA-ARS Vegetable Crops Research Unit, Horticulture Department, University of Wisconsin, Madison, WI 53706, USA

**Keywords:** *Cucurbita moschata*, pumpkin, hull-less, naked seeds, alternative splicing, map-based cloning, bulked segregant analysis, seed development

## Abstract

In pumpkin (*Cucurbita moschata*), the naked or hull-less seed phenotype has great benefits for breeding this crop for oil or snack use. We previously identified a *naked seed* mutant in this crop. In this study, we report genetic mapping, identification, and characterization of a candidate gene for this mutation. We showed that the naked seed phenotype is controlled by a single recessive gene (*N*). The bulked segregant analysis identified a 2.4 Mb region on Chromosome 17 with 15 predicted genes. Multiple lines of evidence suggested that *CmoCh17G004790* is the most probable candidate gene for the *N* locus which encodes a NAC transcription factor WALL THICKENING PROMOTING FACTOR 1 (*CmNST1*). No nucleotide polymorphism or structural variation was found in the genomic DNA sequences of *CmNST1* between the mutant and the wildtype inbred line (hulled seed). However, the cDNA sequence cloned from developing seed coat samples of the *naked seed* mutant was 112 bp shorter than that from the wildtype which is due to seed coat-specific alternative splicing in the second exon of the mutant *CmNST1* transcript. The expression level of *CmNST1* in the developing seed coat was higher in the mutant than in the wildtype during early seed coat development which was reversed later. Transcriptomic profiling with RNA-Seq at different stages of seed development in the mutant and wildtype revealed a critical role of *CmNST1* as a master regulator for the lignin biosynthesis pathway during seed coat development while other NAC and MYB transcription factors were also involved in forming a regulatory network for the building of secondary cell walls. This work provides a novel mechanism for the well-characterized *NST1* transcription factor gene in regulating secondary cell wall development. The cloned gene also provides a useful tool for marker-assisted breeding of hull-less *C. moschata* varieties.

## 1. Introduction

The pumpkin and squash in the genus *Cucurbita* (mainly *C. pepo*, *C. maxima*, and *C. moschata*, 2*n* = 2*x* = 24) are economically important crops worldwide. In addition to their primary uses as vegetables (immature fruit) or staple foods (mature fruit), and ornamentals, their seeds are also consumed as both seed snacks and culinary oil [1,2]. Pumpkin/squash seeds are rich in oil, protein, unsaturated fatty acids, and antioxidants that have many health benefits (e.g., [3,4,5,6,7,8]). One drawback in the use of cucurbit seeds is their thick and leathery seed coat that is hard to decorticate. One way to address this is to use *naked* or *hull-less* seed mutants which lack a complete seed coat, and thus are preferred for snacking and oil production because they eliminate the need for manual de-hulling prior to use. Naked pumpkin seeds are a popular ingredient in many snacks, breads, breakfast cereals, soups, and other edible goods [9,10]. Thus, the development of oil-or snack-use pumpkin/squash varieties is also an important objective for breeding programs [3,11,12].

The first spontaneous *hull-less* mutant called Styrian (hull-less) was reported in *C. pepo* subsp. *pepo* var. Styriaca almost 80 years ago in Austria [3]. Since then, several more *hull-less* mutants have been described in all three major *Cucurbita* crops, which were all shown to be controlled by a single recessive gene *n* (for *naked seed*) or *h* (for *hull-less seed*) [2,12,13,14] although some modifiers having a minor influence on testa development are also possible [3,12]. There were a few early studies on molecular mapping of the *n* or *h* locus in *C. pepo* (e.g., [15,16,17]). More recently, three studies on fine genetic mapping of the *hull-less* mutation in *C. pepo* [18,19,20] suggested that the hull-less seed phenotype in the pumpkin line HLP36 is due to a single recessive gene (*cphl-1*). Linkage mapping identified a candidate gene region that was 2.1 Mbp on chromosome 12 including secondary cell wall and lignin biosynthesis-related transcriptional factors viz., “NAC” (*Cp4.1LG12g04350*) and “MYB” (*Cp4.1LG12g03120*) that were suggested as possible candidates for the *cphl-1* locus. Meru et al. [20] conducted linkage mapping in the segregating populations derived from a cross between two *C. pepo* inbred lines, Kakai (*hull-less*) and Table Gold Acorn (hulled), and identified two SNPs that were significantly associated with the hull-less trait in cultivars and accessions of diverse genetic backgrounds. From this research, several candidate genes were proposed including a *NAC domain-containing protein* gene and a *Fiber Protein fb11* gene involved in lignin accumulation and cell wall deposition across plant species, respectively. By BSA (bulked segregant analysis) and fine mapping, Lv et al. [19] found that mutation of a single gene, *NAC SECONDARY WALL THICKENING PROMOTING FACTOR 1* (*NST1*), accounts for the hull-less trait in the *C. pepo* line P-HL. They further proposed that a 14-bp sequence insertion in the *CpNST1* gene causes premature termination of *CpNST1* translation, leading to a lack of secondary cell wall (SCW) biosynthesis in hull-less seed coats. From these studies, it seems the *hull-less* mutations in three *C. pepo* lines share the same candidate gene (*CpNST1*, a NAC transcription factor gene).

In the regular, hulled seed plants (wildtype or WT hereinafter), during the initial seed coat development, the seed testa exhibits five distinct layers including epidermis (E), hypodermis (H), sclerenchyma (S), parenchyma (P), and innermost chlorenchyma (C) [21,22]. In hull-less seeds, four seed tissue layers (E, H, S, and P) collapse forming a papery thin hyaline hull that may reveal the green color of the underlying C layer [12,21,23,24]. The changes in the seed testa of the mutant are associated with reduced biosynthesis of cellulose and lignin in the secondary cell walls which starts approximately 10–15 days after pollination (DAP) (e.g., [21,22,25]). Lignin deposition diminution also coincides with reduced expression of the genes or enzyme activities in the lignin biosynthesis pathways or secondary cell wall development. For example, the expressions of lignin/cellulose synthesis genes for cellulose synthase (CES), phenylalanine ammonia lyase (PAL), cinnamoyl CoA reductase, 4-coumaric acid CoA ligase (4CL), cinnamyl alcohol dehydrogenase (CAD), cinnamic acid-4-hydroxylase (C4H), glutathione reductase, and abscisic acid-responsive protein E exhibited significantly lower expression in the *C. pepo hull-less* mutant than in the WT [19,25,26]. 

While we now have a better understanding of the genetic and molecular basis of the hull-less mutation in *C. pepo*, the work of such mutants in the other two *Cucurbita* species (*C. moschata* and *C. maxima*) is limited. We previously characterized a *hull-less* seed (thin-coated or naked) *C. moschata* mutant that was first identified in China [13,27]. From the mutant, a hull-less variety (65-1-8) and two hull-less inbred lines were developed with large naked seeds that are rich in fat and proteins with good commercial potential [13,28]. We also investigated the anatomical and biochemical bases of this *hull-less* mutant and found that the degeneration or absence of the typical seed testa structure in hull-less seeds of *C. moschata* is due to the reduced activity of key enzymes in the lignin biosynthesis pathway [25]. However, the genetic basis and underlying candidate gene for this mutation are unknown which hinders its efficient use in breeding pumpkin varieties through marker-assisted selection. Thus, the objectives of the present study were to (1) investigate the inheritance of hull-less seed phenotype in the mutant line (HLS-B); (2) conduct genetic mapping and map-based cloning to identify the candidate gene for the mutant allele (*n* locus); (3) understand the regulatory network for seed coat development in *C. moschata*. Here we report mapping and identification of a candidate gene of the *n* locus through BSA-Seq. We performed RNA-Seq with seed coat samples collected from different development stages to reveal important genes and pathways for seed coat development. We showed the NAC transcription factor *CmNST1* was a candidate gene for the *n* locus, and alternative splicing in the *CmNST1* gene in the seed coat was responsible for the hull-less phenotype. 

## 2. Materials and Methods

### 2.1. Plant Materials and Phenotyping of Seed Coat 

**Figure 1:**
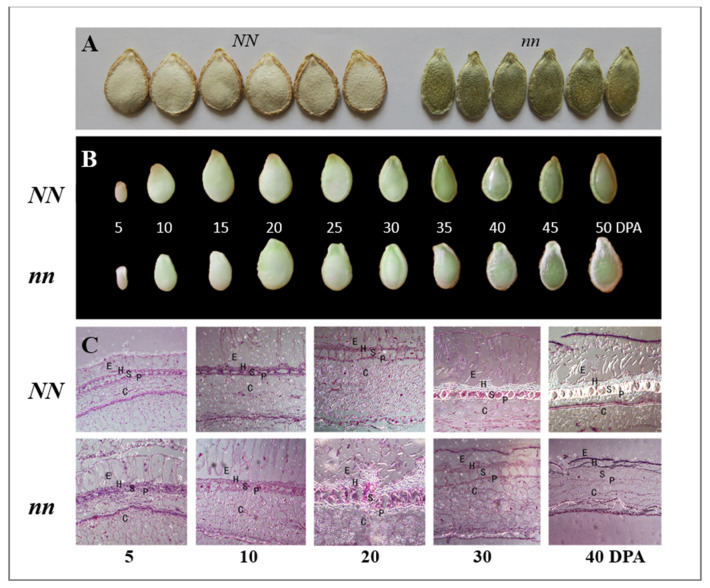
Seed development and scanning electronic microscopic (SEM) observations of seed testa in wildtype (HS-A, *NN*) and *naked seed* (HLS-B, *nn*) pumpkin lines. (**A**) Appearance of mature dry seeds of WT (left) and mutant (right). (**B**) Morphology of fresh seed coat at different development stages in WT and mutant. (**C**) SEM view of seed testa structure in WT and mutant at different developmental stages. E = epidermis, H = hypodermis, S = sclerenchyma, P = parenchyma, C = chlorenchyma. DAP, days after pollination. Scale bars, 25 μm.

The *hulled seed* (HS-A, wildtype or WT hereinafter) and *hull-less* or *naked seed* mutant HLS-B (mutant hereinafter) inbred lines of *C. moschata* (Figure 1A,B) were obtained from the College of Horticulture, Shanxi Agricultural University (SAU), Taiyuan, China. A recombinant inbred line (RIL) population with 189 F_8_ RILs was developed from the cross between the two inbred lines through single seed descent (SSD), which was used for linkage analysis and candidate gene identification. Reciprocal F1’s and additional segregating populations (F_2_, BC_1_P_1_, and BC_1_P_2_) from the same cross were used to investigate the inheritance of the mutant. All plants were grown in plastic greenhouses in the Dongyang Innovation Base of SAU (Jinzhong, China) and grown with trellis support (1.0 m × 0.5 m spacing). Management of plants followed standard local cultivation practices. Seed coat phenotypes were visually assessed on mature seeds as either hulled (WT) or hull-less (naked) which were sampled from fruits at least 45 days after pollination (DAP).

### 2.2. Bulked Segregant Analysis (BSA) and Resequencing (BSA-Seq)

We used BSA-Seq to locate the *naked seed* (*CmN*) mutant allele in a subchromosomal region. Young leaf samples of RILs were collected and kept in a −80 °C freezer. Once the seed coat phenotype of each RIL was determined, two bulks, HS-A and HLS-B, were constructed by pooling equal amounts of leaf samples from 42 WT and 42 mutant RILs, respectively. Genomic DNAs from the two bulks and two parental lines were extracted using the CTAB (cetyl trimethylammonium bromide) method. The four DNA samples were then sent for high throughput resequencing at Biomarker Technologies Corporation (Beijing, China) using the Illumina Hi-Seq^TM^ 2500 platform following service provider’s procedures. High-quality read sequences were aligned to the *C. moschata* reference genome (var. Rifu) (https://www.cucurbitgenomics.org/, accessed on 10 April 2023) by BWA with default parameters [29]. SNP calling between two pools and two parental lines was performed with the GATK pipeline [30]. Homozygous SNPs were employed for calculating SNP index in each sample, and ΔSNP-index was calculated from SNP-index (HS-A bulk) minus SNP-index (HLS-B bulk).

### 2.3. Linkage Mapping and Identification of Candidate Gene for the CmN Locus

BSA-Seq delimited the *CmN* locus into a ~2.2 Mbp region. To narrow the candidate interval, SNPs and polymorphic InDel markers that were detected in both mixed pools and two parental lines in the target region were selected. The SNPs were converted to CAPS or dCAPS for SNP genotyping. Primers design used the dCAPS Finder 2.0 and Primer Premier 5.0 software. Once the polymorphism of a marker was validated between the pools and two parental lines, it was applied to the RIL population. The polymorphic SNPs were used in KASP (Kompetitive allele-specific PCR) genotyping in the RILs. KASP primers were designed with Primer Premier 5.0 (https://primer-premier-5.software.informer.com/, accessed on 10 April 2023). Primer information for all markers used in this study is provided in Supplemental Table S1.

For CAPS/dCAPS assays, the PCR was carried out in a 25 μL reaction containing 2.5 μM MgCl_2_, 0.25 μM of each dNTP, 2 units of *Taq* polymerase (Takara) and 0.5 μM of each primer. For InDel markers, PCR was carried out using 10 μL samples containing ~40 ng of genomic DNA, 0.4 μM of each primer, 400 μM dNTPs, 1× reaction buffer, and 0.5 U *Taq* polymerase (Takara). The PCR amplifications were performed with the following conditions: 94 °C for 5 min; 35 cycles of 94 °C for 30 s, 55–60 °C for 30 s, and 72 °C for 30 s; and a final extension at 72 °C for 5 min. PCR products for indel markers or restriction enzyme-digested PCR products from CAPS assays were resolved via Native PAGE (Native Polyacrylamide Gel Electrophoresis) and visualized with silver staining. 

For KASP assays, the PCR was performed in a BioRad CFX-96 Thermal Cycler with 10 μL reaction mixture containing 1× KASP master mix, 0.17 μM KASP assay mix (two forward allele-specific primers and a common reverse primer), and 20 ng of genomic DNA. PCR was performed with the following cycling conditions: an initial denaturation at 94 °C for 10 min, 10 cycles of denaturation at 94 °C for 20 s, annealing at 61–65 °C for 45 s, 35 cycles of denaturation at 94 °C for 20 s, annealing at 55 °C for 45 s. 

Genotypic data for all markers were scored as A, B, and H representing homozygous WT, mutant and heterozygous genotypes, respectively. Genetic mapping was carried out with the 189 RILs using JoinMap 4.0. The recombination values were converted into map distances (centimorgan or cM) using the Kosambi mapping function. 

### 2.4. Sequence Analysis of CmN Candidate Gene

Linkage analysis suggested that *CmN* is located in a ~390 kb physical interval on *C. moschata* Chr17. Predicted genes in this region were extracted from the *C. moschata* reference genome (http://www.cucurbitgenomics.org/, accessed on 10 April 2023). We also performed manual annotation of this region with FGENESH (http://linux1.softberry.com/berry.phtml/, accessed on 10 April 2023). Genomic DNA, full-length cDNA, and promoter sequences of the candidate gene (*CmNST1*) were cloned from two parental lines and selected RILs. The intron-exon structure was further validated by alignment of transcripts against the cDNA sequence of the candidate gene from public pumpkin/squash transcriptome data.

Since alternative splicing was found to be responsible for the naked seed phenotype in HLS-B, we verified this by cloning of cDNA sequences from different organs and seed coat tissues of WT and mutant RILs at different fruit development stages. For cDNA sequencing, total RNA was extracted using the TransZolUp kit from Transgen Biotech Co., Ltd. (Beijing, China) (https://www.transgenbiotech.com/, accessed on 10 April 2023). cDNA was synthesized with GoScript™ Reverse Transcription kit from Promega Co. (Madison, WI, USA) (https://www.promega.com.cn/, accessed on 10 April 2023). 

The predicted genomic DNA and mRNA sequences of *C. moschata* reference genome (cv Rifu), its homologs in *C. maxima* and *C. pepo* were extracted from the cucurbit genome database (https://cucurbitgenomic.org/, accessed on 10 April 2023), which were aligned with gDNA and cDNA sequences of HS-A and HLS-B obtained from the present study using Clustal W2 (https://www.ebi.ac.uk/Tools/msa/clustalw2/, accessed on 10 April 2023). 

### 2.5. Quantitative Real-Time PCR (qPCR)

For expression analysis of candidate gene, equal amounts of seed coat samples at 10, 20, 30, and 40 DAP of five WT and five mutant RILs were pooled. Total RNA was extracted by the TRIzol method (Invitrogen, Carlsbad, CA, USA). cDNA was synthesized using GoScript™ Reverse Transcription Mix (Promega, Madison, WI, USA). qPCR was performed using TranStart^®^Tip Green qPCR Super Mix under the following conditions: initial denaturation at 94 °C for 30 s, followed by 45 cycles of denaturation at 94 °C for 5 s, annealing at 60 °C for 31 s, and final extension at 60 °C for 31 s. There were three biological replicates for each sample. The *actin* gene was used as the internal reference. Relative expression levels were quantified using the 2^−ΔΔCT^ method [31], and significance analysis was performed using SPSS 23.0 software.

### 2.6. Bulked Segregant RNA-Seq (BSR-Seq)

We investigated the transcriptomes of WT and mutant bulks from RILs with RNA-Seq. Fresh seed coat tissues were collected from F_8_ RILs at 10, 20, 30, and 40 DAP. At each time point, the WT and mutant pools were constructed by pooling equal amounts of seed tissues from five WT and naked seed mutant RILs, respectively. Each pool had three biological replications. Total RNA for the 24 bulks was extracted using RNAprep Pure Plant Kit (Tiangen Co., Beijing, China). cDNA library preparation and high throughput Illumina sequencing was performed in Biomarker Biotechnology Inc. following service provider’s protocols. After initial data quality assessment and filtering, Spearman’s correlation coefficients were calculated to evaluate reproducibility among three biological replicates of each sample. The clean reads filtered from raw data were mapped onto the *C. moschata* reference genome (http://cucurbitgenomics.org/, accessed on 10 April 2023) with HISAT2 [32]. Assembly of mapped reads was performed with StringTie [33]. After filtering low-quality reads (unknown nucleotides > 5% or low Q value ≤ 20%), FPKM (fragments per kilobase of transcript per million mapped reads) values were calculated to estimate gene expression levels by Cufflinks software. Differentially expressed genes (DEGs) between the WT and mutant at each time point were determined using the DESeq [34], and the false discovery rate (FDR) ≤ 0.05 and |log2(fold change)| ≥ 1 were used as the thresholds to determine statistically significant differences in gene expression. The DEGs at 10 DAP between the mutant and WT were further subjected to various enrichment analyses including GO (Gene Ontology) analysis, COG (Cluster of Orthologous Groups of proteins) analysis, KEGG (Kyoto Encyclopedia of Genes and Genomes) pathway analysis. Finally, SNPs and Indels (insertion-deletion) between mutant and WT in the transcripts from RNA-Seq were detected with GATK4 [30] and annotated with SnpEff [35].

## 3. Results

### 3.1. Phenotypic Characterization and Inheritance of Naked Seed Mutation

In multiple years of observations, the *naked* or *hull-less* seed mutant line (HLS-B) always exhibited a thin layer of seed coat on the seed kernel, and the wildtype (WT) line had a thick seed coat (Figure 1A). No other visual differences could be seen in the developing seeds at different development stages (Figure 1B). 

We observed seed coat development in the mutant and WT under a scanning electron microscope (SEM) (Figure 1C). The seed testa of WT seeds gradually formed five clearly distinguishable tissue layers including from outside to inside the epidermis (E), hypodermis (H), sclerenchyma (S), parenchyma (P), and chlorenchyma (C). The five-layer cell wall in the WT started thickening at approximately 20 days after pollination (DAP) by lignin deposition, especially in the S layer until seed maturity at ~50 DAP. In the mutant, although the five-layer structure was also formed in the seed testa at ~20 DAP, lignin deposition in the testa was lacking, and the successive degradation of the five-layer structure was evident (Figure 1C). These observations suggest that the naked seed mutant is probably defective in lignin biosynthesis and secondary cell wall formation.

To analyze the inheritance of the naked seed phenotype, we examined the segregation of this trait in populations derived from the cross between WT and HLS-B inbred lines. The F_1_’s from mutant (female) × WT (male) (*n* = 283) and from WT (female) × mutant (male) (*n* = 272) all had naked and hulled seeds, respectively. All 253 BC_1_P_1_ plants displayed the WT hulled seed phenotype. Among 228 BC_1_P_2_ plants, 116 and 112 were hull-seeded and had naked seeds, respectively, corresponding to the expected 1:1 segregation ratio (χ^2^ = 0.317, *p* = 0.852). Among 165 F_2_, there were 121 hull-seeded and 44 hull-less seeded individuals, which was consistent with a 3:1 segregation ratio (χ^2^ = 0.317, *p* = 0.188). Finally, of the 189 F_8_ RILs, 96 and 93 exhibited hulled seeds, and hull-less seeds, respectively, fitting a 1:1 tested ratio in the χ^2^ test (*p* = 0.8272). These data were consistent with previous studies on the inheritance of *naked seed* phenotype in different pumpkin/squash varieties (see Introduction) that the *naked seed* mutation is controlled by a single recessive gene, which was designated as *n* (for *naked seed*) per gene nomenclature rules in pumpkin/squash [14,36]. 

### 3.2. BSA-Seq Analysis 

**Figure 2:**
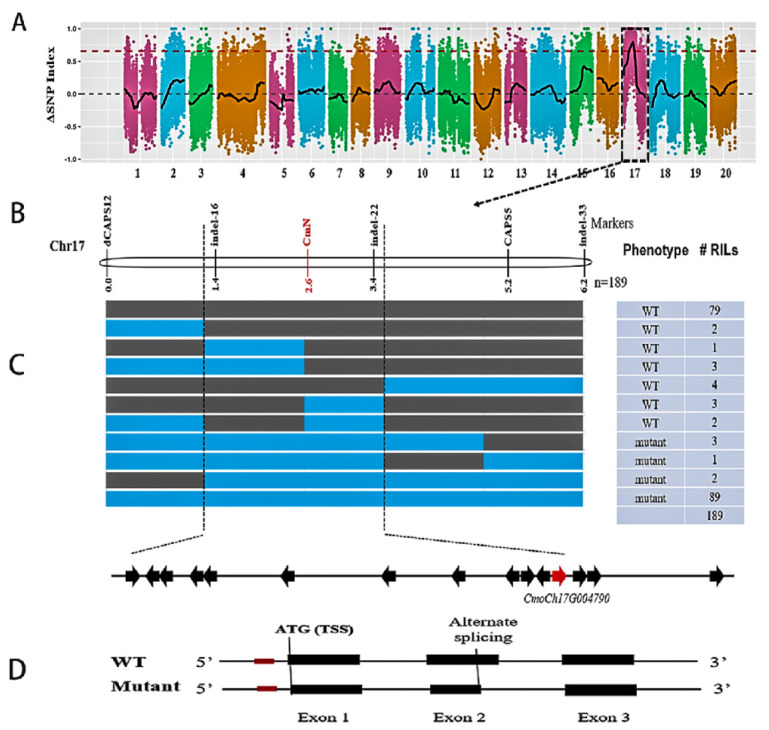
Identification of candidate gene for the *N* locus in *C. moschata*. (**A**) Genomewide ΔSNP-index plot using SNPs between the mutant and WT bulk shows that the *N* locus is located in a 2.42 Mbp region on Chr17. (**B**) Linkage mapping in RILs narrowed down the *N* locus into a 389.5-kb region with 15 predicted genes. (**C**) Multiple lines of evidence support *CmoCh17G004790* as the most possible candidate gene for the *N* locus (Red in (**C**)). (**D**) Sequence analysis indicates that the *naked seed* mutation is due to an alternative splicing in seed coat in the mutant. In (**A**), the *X*-axis shows chromosomes. The *Y*-axis is ΔSNP-index value. Red line is 99% significance threshold. In (**B**), the grey and blue bars represent different haplotypes defined by molecular markers. The genotype and # of RILs carrying each haplotype in the RIL population are shown to the right. In (**D**), the red block upstream of the translation start site (TSS) indicates SSR (simple sequence repeat) that was proposed to be the causal variant of naked seed in *C. pepo* [19].

Mutant and WT bulks were constructed from 42 mutant and 42 WT RILs, respectively. Illumina sequencing of the two bulks and two parental lines generated ~27.47 Gb clean reads (Q30 > 80%) including 11.07 Gb from the parents and 16.4 Gb from the two bulks. The main statistics of BSA-Seq are presented in Supplementary Table S2. The average sequencing depths for the two parents and the two F_8_ RIL pools were 13.5× and 21×, respectively. These reads were mapped onto the *C. moschata* reference genome with an average mapping rate of 97.7%. From 1,019,929 SNPs and small Indels detected, after serial filtering processes (biallelic, single copy, read depth > 4, consistent between two bulks and two parental lines), 377,185 high-quality SNPs were used for calculating ΔSNP/Indel-index and the Euclidean distance (ED) estimation. The genome-wide ΔSNP-index plot is shown in Figure 2A. The ED plot is illustrated in Supplementary Figure S1. The ΔSNP-index association analysis mapped the *N* locus to a 2.38 Mbp physical interval on Chromosome 17 (2,810,000 bp to 5,190,000 bp) in the reference genome. BSA with Indels and ED resulted in a slightly larger interval that overlapped with the one identified with the ΔSNP-index method suggesting the *N* locus is located in this region.

### 3.3. Linkage Mapping and Candidate Gene Identification for the N Locus

To narrow down the candidate gene region, we developed new molecular markers in the 2.38 Mbp region. From BSA seq, 10,433 SNPs and 2434 Indel polymorphisms were identified in this region, from which 59 non-synonymous SNPs and 47 Indels were targeted for primer design. Both CAPS and KASP assays were employed for SNP genotyping. Polymorphisms of these markers were first verified between the two bulks and parental lines, then applied in the RIL population. Finally, three Indels and two KASP/CAPS markers were successfully mapped in the RIL population. Primer information of these mapped marker loci is shown in Supplementary Table S1. The resulting linkage map and nine haplotypes defined by these markers among 189 RILs are presented in Figure 2B. Through linkage analysis, the *N* locus was placed into a 389.5-kb region delimited by two flanking markers indel-16 and indel-22.

Fifteen genes (#1–15) were annotated in the *C. moschata* reference genome (Figure 2C), which are listed in Supplementary Table S3. To identify a potential candidate gene for the *N* locus, we first examined the sequence variation in this region between two parental lines. From BSA-seq, 1121 SNPs and 28 Indels were identified against the reference genome. The complete list of the 1159 variants is provided in Supplementary Table S4. Many SNPs/Indels were polymorphic between the WT or mutant and the reference genome, but not between the WT and the mutant. We scanned all these variants to find the association of nucleotide variation with the *naked seed* vs. *hulled seed* phenotypes using the following criteria: high quality (nucleotide accuracy and coverage), polymorphic between WT and mutant, located in promoter or coding regions, and non-synonymous mutations. As the line of the reference genome is also a wildtype, any causal SNP must also carry the same allele as the WT/HS-A line used in this study. When a polymorphism was located inside an exonic region, we further examined its variation in cDNA sequences extracted from the RNA-Seq data (see below). None of 1159 SNPs/Indels met these criteria suggesting the causal polymorphism may not be present at the genomic DNA (gDNA) level. 

**Figure 3:**
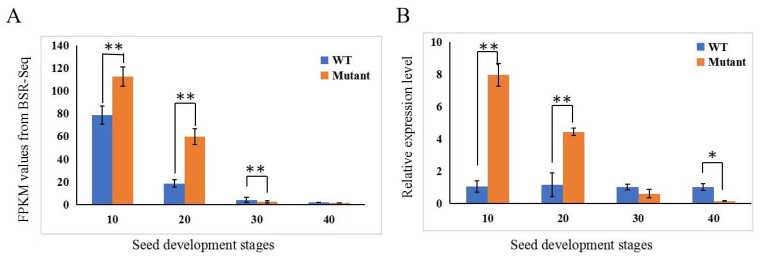
Expression of *CmNST1* in seed coat from RNA-Seq (**A**) and qPCR (**B**). The expression level of the *CmNST1* at 10, 20, 30, and 40 DAP from BSR-Seq is expressed in FPKM (fragments per kilobase of transcript per million mapped reads) values. ** *p* < 0.01; * *p* < 0.05. Error bar represents mean ± SD (*n* = 3 biological replications).

We further investigated the expression dynamics of the 15 genes from the RNA-Seq data (see below for more details). FPKM (fragments per kilobase of transcript per million mapped reads) values were extracted from WT and mutant samples at four time points (10, 20, 30, and 40 DAP, three biological reps each), which are provided in Supplementary Table S3. The bar graphs of FPKM values are plotted and illustrated in Figure 3A (for Gene #12), and Supplementary Figure S2 (the remaining 14 genes). We calculated the log2 (fold change) (WT vs. mutant) value for each gene, which is also presented in Table S3. Among all 15 genes, only the 12th gene, *CmoCh17G004790* that was predicted to encode NAC domain-containing protein 43 (NAC43) showed a statistically significant difference in expression between the mutant and WT at 30 DAP using the criteria of FDR < 0.05 and |log2(FC)| ≤ 1. The expression dynamics of *CmoCh17G004790* in the developing seed coat of the two lines were further validated by qPCR, which is presented in Figure 3B. Its expression was higher in the seed coat of the mutant than in the WT at 10 and 20 DAP, which showed the opposite at 30 and 40 DAP. *CmoCh17G004790* is a homolog of *Arabidopsis* gene *AT2G46770* that encodes a NAC transcription factor WALL THICKENING PROMOTING FACTOR 1 (NST1) [37]. In *C. pepo*, its homolog *CpNST1* has been shown to be the candidate gene for the *hull-less* seed mutation [19]. These data suggested that *CmoCh17G004790* (*CmNST1* hereinafter) is a possible candidate for the *N* locus in *C. moschata*.

### 3.4. Sequence Analysis Suggests Alternative Splicing in the CmNST1 Transcript May Contribute to Defective Seed Coat Development in the Mutant 

**Figure 4:**
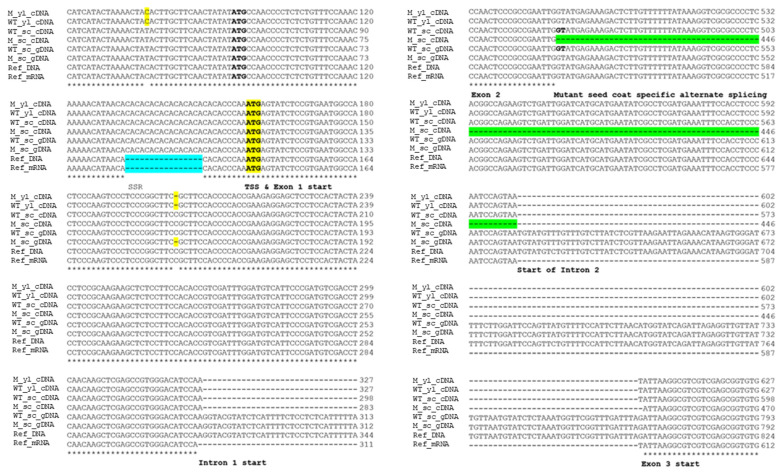
Alignment of genomic DNA (gDNA) and cDNA/mRNA of part of the *CmNST1* gene showing translation start (TSS) and alternative splicing (AS) in the seed coat of *naked seed* mutant. The SSR in 5′ UTR and the AS site in the second exon are highlighted in blue and green colors, respectively. The proposed start codon (ATG, boldface typed) by Lv et al. [19] and current study (boldface typed and highlighted in yellow) is also shown. M = mutant (*nn*), WT = wildtype (*NN*), yl = young leaves, sc = seed coat, Ref = reference genome (Rifu from https://cucurbitgenomics.org/, accessed on 10 April 2023).

Data from BSA-Seq identified no causal polymorphisms in gDNA sequences between the mutant and WT in any of the 15 genes. With Sanger sequencing, we further cloned the gDNA and cDNA sequences of *CmoCh17G004790* from the different organs/tissues in the mutant and WT including young leaves, root, stem at seedling stage, seed coat of developing seeds at 10, 20, 30, and 40 DAP and leaf tissues from adult plants. The gDNA and cDNA sequences and their alignment from the seed coat and young leaves together with the gDNA and mRNA sequences from the reference genome (cv Rifu) are shown in Supplemental File S1. The alignment of part of the gDNA and cDNA sequences of *CmNST1* in WT, mutant, and the reference genome is presented in Figure 4. Annotation of this gene in the *C. moschata* genome suggested three exons and two introns of *CmNST1* (Figure 2D; Supplemental File S1), which was consistent with the cloning of cDNA sequences and manual annotation, as well as that in *Arabidopsis*. Consistent with BSA-Seq, no sequence variation at the gDNA level was found between WT and the mutant. However, a 112 bp deletion was found in the cDNA cloned from seed coat tissue as compared with cDNA cloned from young leaves, which started from inside the second exon and ended at the start of the second intron (Figure 4). We wondered if this was due to a sequencing error and therefore we cloned gDNAs and cDNAs from different organs at different development stages including cotyledons, root, stem, and young leaves from seedlings, as well as seed coat samples collected at 10, 20, 30, and 40 DAP in both the mutant and WT. In each case, multiple clones were Sanger sequenced, and the results were the same. That is, all cDNA clones from the seed coat of the mutant at any time point had the 112 bp deletion as compared with cDNA from vegetative organs of the mutant and any organs/tissues from the WT or the reference genome (Supplemental File S1) supporting alternative splicing (AS) in the developing seed coat of the mutant may be associated with the *naked seed* mutation. 

We also compared the gene structure and annotation of the *CmNST1* homolog genes in *C. pepo* (*Cp4.1LG12g04350*) and *C. maxima* (*CmaCh17G005080*). The gDNA and mRNA sequences of *C. maxima* and *C. pepo* were downloaded from the cucurbit genomics database. The sequences and their alignment among the three species are shown in Supplemental File S2. The gDNA and mRNA sequences from the *C. pepo* reference genome were shorter than the other two homologs including missing the translation start codon. Only two exons were annotated in the *C. pepo* gene which may represent a misannotation of *Cp4.1LG12g04350*. Lv et al. [19] suggested that the *hull-less* seed mutation in *C. pepo* is due to an insertion of a 16 bp sequence in the first exon of *CpNST1* (*Cp4.1LG12g04350*). We found that this was a simple sequence repeat (SSR) with a CA motif which was presented in both the mutant and WT of *C. moschata* of our study (Figure 4) thus excluding it as a possible contributing mutation to *naked seed* in HLS-B inbred. Interestingly, in *C. moschata*, the SSR was 5bp upstream of TSS of *CmNST1* whereas, in *C. pepo*, the start codon (ATG) was proposed to be ~60 bp upstream of the second proposed start codon in *C. moschata* ([19]; also Figure 4). While this discrepancy needs further clarification, these observations may suggest a different mechanism associated with *naked seed* mutation in *C. pepo* [19] and *C. moschata* (this study).

### 3.5. Transcriptome Profiling Reveals Regulatory Gene Network for Seed Coat Development in C. moschata

To understand the gene regulatory network controlled by the *CmNST1* gene, we conducted BSR-Seq to study the transcriptomes of the seed coat of the mutant and WT. Seed coat samples were collected from developing seeds at four stages: 10, 20, 30, and 40 DAP with three biological replications per sample (total of 24). Main RNA-seq statistics are presented in Supplemental Table S5. The complete transcriptome datasets have been deposited into the CNGB Sequence Archive (CNSA) of the China National GeneBank Database (CNGBdb) with accession number CNP0003716. Nearly 93.4% of bases in each sample had a Q-score no less than Q30. Of the ~510 million high-quality paired-end reads (~152 Gbp) generated from 24 cDNA libraries, ~92.5% of reads could be mapped to the *C. moschata* reference genome (Supplemental Table S5). The high quality of the RNA-Seq datasets could be seen from the high Spearman’s rank correlation coefficient plot among replicates of each sample (Supplemental Figure S3). We explored SNPs and Indels in transcripts between WT and mutant from BSR-Seq. Consistent with results from BSA-Seq and Sanger sequencing results, no polymorphisms were detected in cDNAs in any of the 15 genes in the 389.5 kb candidate gene region further confirming that *CmNST1* was the most likely candidate for the *n* locus. In addition, as compared with the reference genome, 2073 new genes not present in the reference genome were annotated. However, the annotation of the 15 genes in the target 389.5 kb region remained the same. 

**Figure 5:**
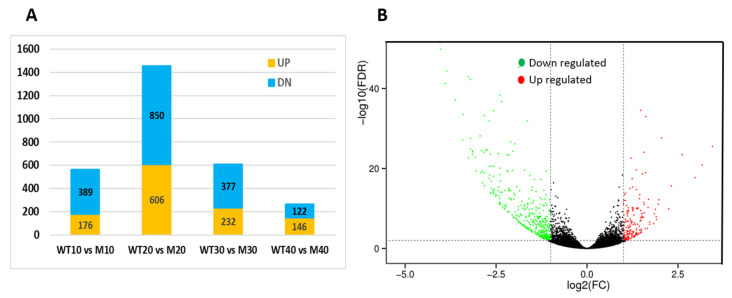
Differentially expressed genes (DEGs) between WT and mutant seed coat transcriptomes revealed with BSR-Seq. (**A**) Bar graphs show number of DEGs in WT vs mutant (M) seed coat transcriptomes at 10, 20, 30, and 40 days after pollination (DAP). DN = down-regulated and UP = up-regulated for genes in mutant as compared with those in WT at threshold of log2(fold change) ≥ 1 and FDR < 0.05. (**B**) volcano plot shows DEGs between WT and mutant at 10 DAP M vs. WT. Green dots are down-regulated genes, while red dots are up-regulated ones, and black dots are genes without significant differences.

Differentially expressed genes (DEGs) were identified using FDR ≤ 0.05 and |log2(fold change)| ≥ 1 as the criteria in various comparisons. Total numbers of DEGs and up- or down-regulated genes in each comparison are presented in Supplementary Table S6. Specifically, 565, 1456, 609, and 268 DEGs were identified between the WT and the mutant at 10, 20, 30, and 40 DAP, respectively. Among them, 176, 606, 232, and 146 DEGs showed higher expression (up-regulated), and 389, 850, 377, and 122 showed lower expression (down-regulated) in the mutant than in the WT seed coat, respectively, which are illustrated in Figure 5A. These data indicated that significant changes in the mutant transcriptomes occurred in early seed coat development, and more genes were down-regulated than those up-regulated. 

Since the WT and mutant showed significant differences in seed coat development that were observed at a very early stage, we focused on DEGs at 10 DAP for further analysis. Of the 565 DEGs at this time point, 429 had been functionally annotated with predicted functions. The gene IDs, predicted functions, GO, COG, and KEGG terms, and their expression dynamics at four seed development stages of the 429 genes are provided in Supplemental Table S7. The candidate gene *CmNST1* and 25 additional DEGs in the lignin biosynthesis pathway were also included in Supplemental Table S7 (total 455). Of the 455 genes, at 10 DAP, many more genes (313 or 68.8%) showed lower expression in the mutant; only 142 (31.2%) had higher expression in the WT. This could clearly be seen from the volcano plot of these DEGs shown in Figure 5B. 

**Figure 6:**
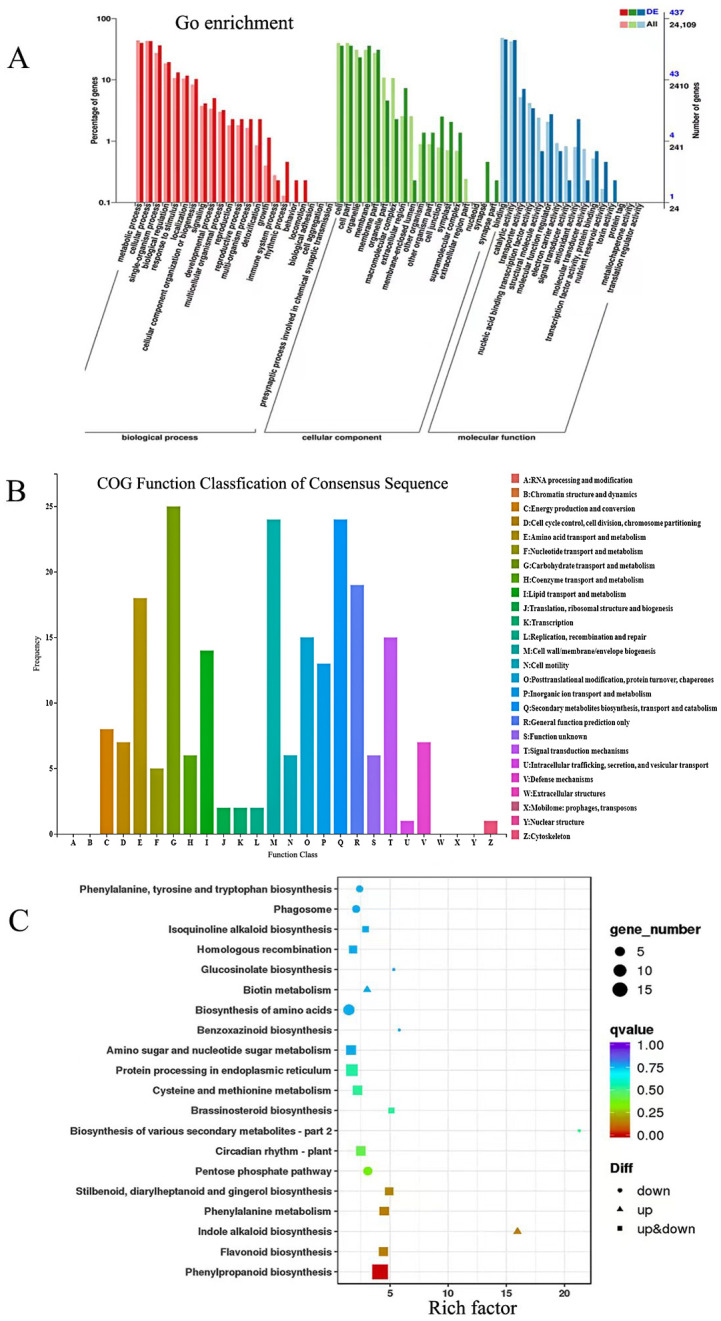
Transcriptome profiling in the WT and mutant pumpkin seed coat with DEGs at 10 days after pollination. (**A**) GO enrichment analysis of DEGs between WT and mutant seed coats. BP, biological process; CC, cellular component; MF, molecular function. (**B**) COG (Cluster of Orthologous Groups of proteins) function classification analysis of DEGs. (**C**) KEGG pathway enrichment analysis of DEGs.

We performed GO enrichment analysis on 429 DEGs between the HS-A and HLS-B at 10 DAP (Figure 6A). The most enriched GO terms in ‘Biological Process’ included metabolic processes, cellular processes, and biological regulation. The most enriched ‘Molecular function’ terms were binding, catalytic activity, transporter, and transcriptional factor activity. COG (Cluster of Orthologous Groups of proteins) function classification analysis of these DEGs (Figure 6B) revealed that the top functional groups were carbohydrate transport and metabolism, cell wall/membrane/envelope genesis, secondary metabolite biosynthesis, transport, and catabolism, as well as signal transduction mechanisms. In KEGG pathway enrichment analysis, the top enriched pathways associated with these DEGs included phenylpropanoid biosynthesis, flavonoid biosynthesis, and phenylalanine metabolism (Figure 6C). These data strongly suggested that genes for enzymes of secondary metabolism, transcription factors, and phytohormones are involved in seed coat development in pumpkin. 

**Figure 7:**
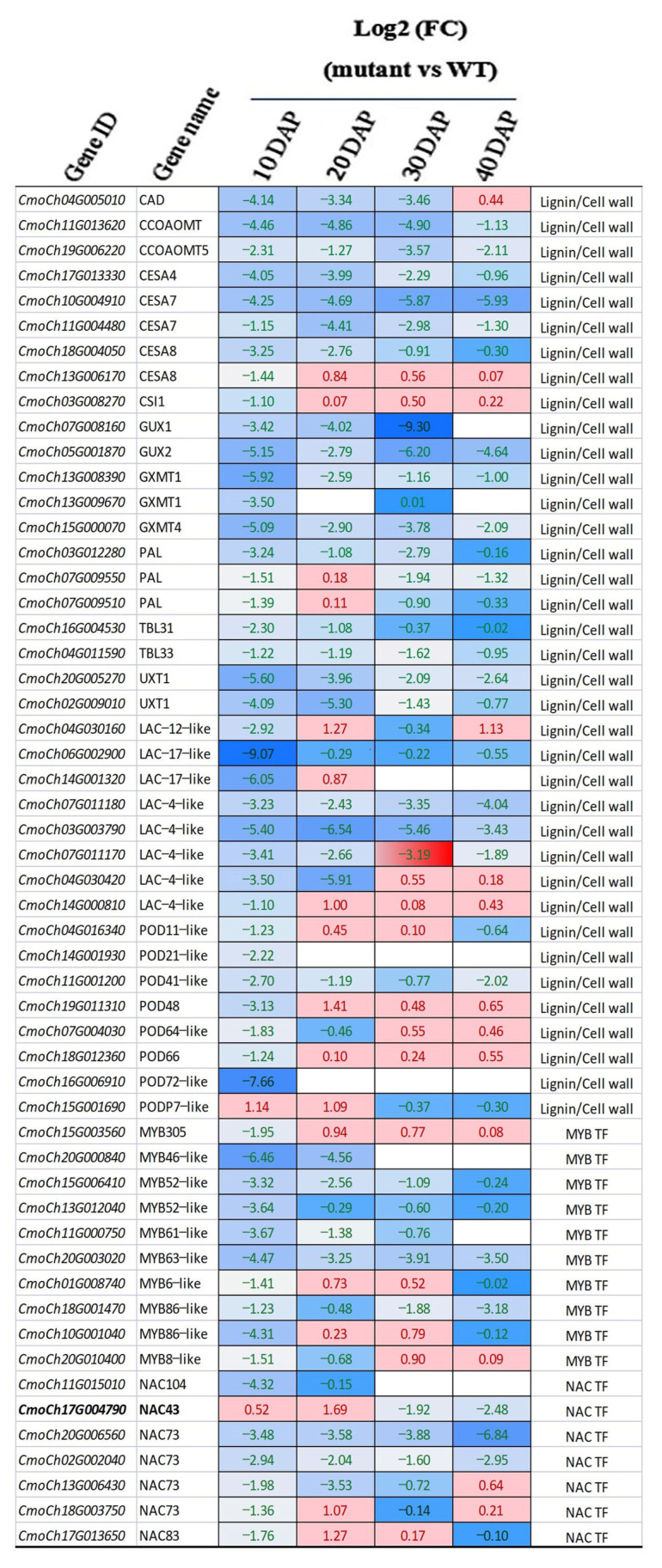
Heatmap of expression level of selected genes involved in lignin biosynthesis pathway or secondary cell wall formation. Value in each cell is log2 (fold change) in the mutant in comparison with the WT from RNA-seq data. The boldface types gene is the candidate for the *n* locus. Cool (light blue, blue) and warm (pink/red) colors indicate down- and up-regulated genes in the mutant as compared to the WT, respectively. See Table S7 for full names of genes.

**Figure 8:**
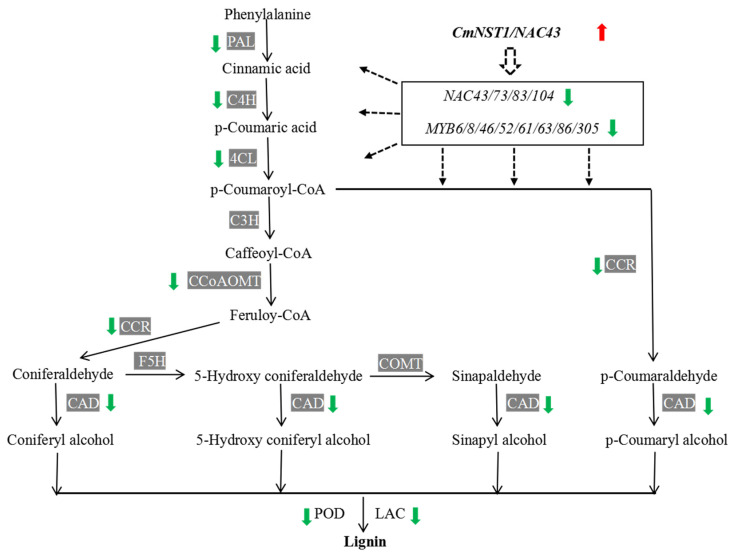
Roles of *CmNST1* in regulating key genes in the lignin biosynthesis pathway and their expression in the *naked seed* (*nn*) mutant as compared with its level in the WT of *C. moschata* from this study. NAC and MYB transcription factor (TF) genes showing differential expression in the two lines are also listed. Dashed arrows indicate the hierarchical network in which *CmNST1* acts as the master regulator that activates other NAC or MYB TFs, which in turn regulate genes in the lignin biosynthesis pathway. Green and red arrows indicate the gene is down- or up-regulated in the mutant as compared with that in the WT. PAL, phenylalanine ammonia lyase; C4H, cinnamate 4-hydroxylase; 4CL, 4-hydroxycinnamoyl-CoA ligase; C3H, p-coumarate 3-hydroxylase; CCoAOMT, caffeoyl-CoA O-methyltransferase; CCR, cinnamoyl-CoA reductase; F5H, ferulate 5-hydroxylase; COMT, caffeic/5-hydroxyferulic acid O-methyltransferase; CAD, cinnamyl-alcohol dehydrogenase; POD, peroxidase; LAC, laccase. The pathway diagram follows [40,41,42].

In our previous study, we found a continued decrease of lignin content in the testa of the mutant but a continued increase in the WT during seed development [25]. We also found that the activities of several enzymes in the lignin biosynthesis pathway were significantly lower in the mutant than in the WT, which included the phenylalanine ammonia lyase (PAL), 4-coumaric acid coenzyme A ligase (4CL), cinnamate-4-hydroxylase (C4H), and cinnamyl alcohol dehydrogenase (CAD). We inferred that the degeneration or absence of the typical seed testa structure in the mutant was due to the reduced activity of key enzymes in the lignin biosynthesis pathway [25]. Lignin is an important component of the secondary cell wall during seed coat development. The biosynthesis of lignin is tightly controlled by a complex regulatory network containing many transcription factors especially members of the NAC and MYB families, as well as phytohormones (reviewed in [38,39,40,41,42]; also see Introduction and Discussion sections). We manually examined all DEGs in the transcriptomes of the mutant and WT at 10 DAP and DEGs of the lignin biosynthesis pathway at 20, 30, or 40 DAP (Table S7). Among the 455 DEGs, 85 (18.7%) encoded enzymes that were directly involved in the lignin biosynthesis pathway or secondary cell wall formation; 45 (10%) were transcription factor genes including seven members of the NAC TF family (NAC43/73/83/104) and 10 members of the MYB TF family (MYB 6/8/46/52/61/63/86/305); 16 were involved in phytohormone biosynthesis/signaling (auxin/IAA, ethylene, BR, GA, and JA) (Table S7). A heatmap of the expression level of 54 selected genes from 85 DEGs involved in the lignin biosynthesis pathway or secondary cell wall formation in terms of log2 (fold change) at the four time points of seed coat development is illustrated in Figure 7. The majority of these genes were down-regulated in the *naked seed* mutant as compared with hulled seed WT. We specifically examined the key genes/enzymes in the lignin biosynthesis pathway which is presented in Figure 8. Most genes-encoding enzymes in this pathway showed reduced expression in the mutant. Some examples were genes for PAL, 4CL, C4H, CAD, CCoAOMT (caffeoyl-CoA O-methyltransferase), and CCR (cinnamoyl-CoA reductase). Multiple members in POD (peroxidases) and laccase (LAC) gene families that play important roles in the last step of lignin biosynthesis were also down-regulated in the mutant (Figure 8). 

Taken together, transcriptome profiling in the mutant and WT during seed coat development clearly supported that the lignin biosynthesis pathway and secondary cell wall formation were compromised in the HLS-B, which was likely due to the alternative splicing mutation in the *CmNST1* (*n* or *NAC3*) gene transcript. Many other transcription factors and hormone biosynthesis/signaling pathways were also involved in the regulation of the secondary cell wall development in *C. moschata*. 

## 4. Discussion

### 4.1. CmNST1 Is a Candidate Gene for the Naked Seed (n) Locus

In this study, we show that the naked seed phenotype in HLS-B *C. moschata* inbred line was controlled by a single recessive gene (*n*). BSA-Seq and linkage analysis placed the *N* locus into a 389.5 kb region on Chromosome 17 containing 15 predicted genes (Figure 2; Table S3). We suggest *CmoCh17G004790* as the most possible candidate gene for the *N* locus which encodes a transcription factor (NAC domain-containing protein 43 or NAC43). Our conclusion was based on multiple lines of evidence. First, from BSA-Seq and resequencing data, no polymorphisms were detected in the coding region or promoter region in any of the 15 genes between the two parental lines, and no consistent haplotypes defined by SNPs or Indels were associated with the phenotypes (the WT of HS-A, Rifu vs. HSL-B mutant) (Table S4). From both BSR-Seq and qPCR, among the 15 genes, *CmoCh17G004790* is the only one showing differential expression between the WT and the mutant (Figure 3; Table S3). More importantly, we found alternative splicing in the mRNA of *CmoCh17G004790* that only occurred in the developing seed coat of the mutant but not in other organs or tissues of the mutant, nor in the mRNA from any tissues or organs of the WT (Figure 4; Supplemental File S1).

*Hull-less* or *naked seed* mutants have been identified in all three *Cucurbita* species, which were all controlled by a single recessive gene (*n* or *h*) [2,12,13,14]. The *n* locus in *C. pepo* has been fine-mapped, and the results from three independent studies all support the NAC transcription gene *Cp4.1LG12g04350* (*CpNST1*) on Chromosome 12 of *C. pepo* as the most possible candidate gene [19,20,43]. *CmoCh17G004790* (*CmNST1*) and *Cp4.1LG12g04350* (*CpNST1*) are homologs in the *C. pepo* and *C. moschata* genomes (Supplemental File S2). Thus, our study represents the first report of cloning of a candidate gene for the *hull-less* mutation in *C. moschata*. Although there is no report on cloning of the *hull-less* locus in *C. maxima*, the data from the present work may suggest that the hull-less seed phenotypes in the three *Cucurbita* species probably share a similar genetic basis and common candidate gene.

The causal mutations in these mutants may be different though. Lv et al. [19] proposed that a 14-bp insertion in *CpNST1* was the causal polymorphism in the *hull-less C. pepo* mutant which would result in premature termination of *CpNST1* translation. The 14 bp sequence includes AA followed by six repeats of a dinucleotide (CA) SSR motif: ((CA)_6_). In the mutant and WT lines used in this study and the *C. moschata* reference genome, the SSRs were (CA)_11_, (CA)_11,_ and (CA)_3_, respectively (Figure 4). This suggests a variation of this SSR in natural populations of *Cucurbita* species although the scope of this variation and its association with the hull-less phenotype requires further investigation. This observation also eliminates the possibility that the SSR is the causal mutation for the hull-less phenotype in the HLS-B inbred line of this study. Another interesting difference is the translation start position in CpNST1 and CmNST1. There are two start codons (ATG) before and after the SSR (Figure 4; Supplemental File S2). Which is the actual TSS may need additional studies.

One common feature between *CpNST1* and *CmNST1* ([19] and this study) was the expression dynamics in the mutant and WT. In this study, *CmNST1* showed higher expression in the seed coats of the mutant than in the WT up to 20 DAP. After that, its expression at 30 and 40 DAP was lower in the mutant than in the WT (Figure 7). The higher expression in the mutant than in the WT was consistent with that in the *C. pepo hull-less* mutant [19] although its expression after 20 DAP was not reported in the early study in *C. pepo*. The higher expression of *NST1* homologs in the mutant was also observed in *Medicago truncatula* [44] supporting a conserved function of *NST1* as a negative regulator in secondary cell wall/seed coat development (see more discussions below).

### 4.2. Alternative Splicing Contributes to Naked Seed Mutation in C. moschata

We found that the *CmNST1* cDNA cloned from seed coat tissues of the mutant was 112 bp shorter than that from the WT suggesting alternative splicing (AS) in the mutant. No AS was found in cDNA sequences of this gene cloned from vegetative organs (root, stem, cotyledon, young and old leaves) of the mutant, nor from cDNAs from any organs or tissues in the WT (Figure 4; Supplemental File S1). This suggests that the AS of *CmNST1* only occurs in the developing seed coat of the *hull-less* mutant. This represents a different mechanism from *C. pepo* [19] for the *hull-less* mutation despite the mutants in both crops having the same candidate gene.

AS refers to the generation of multiple splice isoforms (mRNA transcripts) from a single gene due to different splicing sites which is a critical mechanism for gene expression regulation at the post-transcriptional level that significantly expands the coding capacity of genomes and improves transcriptome plasticity and proteome diversity [45]. AS is a common phenomenon in plants that plays an important role in plant adaptation and evolution. For example, early studies estimated that at least 42% *Arabidopsis* and 48% rice intron-containing genes are alternatively spliced [46,47]. In silico analysis of transcriptomic data from high throughput transcriptome sequencing suggested > 70% of plant genes may have AS in a tissue-specific, developmental, or signal transduction-dependent manner (e.g., [48,49,50]). AS in many genes playing important roles in plant growth and development and adaptation to environments has been documented (reviewed in [51]). To the best of our knowledge, AS has not been reported in any genes that play a role in seed coat development.

Several types of AS have been observed which include exon skipping (ES), intron retention (IR), alternative 5′ splice site (alt 5′SS), alternative 3′ splice site (alt 3′SS), variable first exon (VFE), variable last exon (VLE) and mutually exclusive exons (MEE) [52,53,54]. In the present study, AS occurred in the second exon of *CmNST1* (Figure 4; Supplemental File S1), which could be classified as an alternative 5′ splice site. Both the mutant and WT seem to fit the GT-AG rule (Figure 4). The reason why the splicing site of the second intron in the mutant started from the first GT is unknown. How does the 112-bp shorter isoform of the mutant transcript result in reduced lignin biosynthesis and secondary cell wall formation, thus a defective seed coat development is an interesting question that merits additional studies.

### 4.3. CmNST1-Regulated Seed Coat Development Involves a Complex Regulatory Network for Lignin Biosynthesis and Secondary Cell Wall Formation

We show that *CmNST1* is a candidate gene for the *n* locus in *C. moschata* which is a homolog of *Arabidopsis NAC SECONDARY WALL THICKENING PROMOTING FACTOR 1* (*NST1*). Plant secondary cells are the building blocks for seed coat development, which are composed primarily of cellulose, lignin, and hemicelluloses. The biosynthesis of cellulose, xylan, and lignin is under elegant transcriptional regulation [55,56,57]. Many members in the NAC transcription factor families have been identified as master regulators by activating different layers of downstream secondary wall-related TFs such as MYBs [44,55,58]. For example, in *Arabidopsis*, important NAC master TF regulators may include NST1/NST2/NST3, VND6 (VASCULAR-RELATED NAC-DOMAIN 6 (VND6) and VND5 (reviewed in [39]). In this hierarchical network, NACs target MYB TFs such as MYB46 and MYB83, which in turn will activate the expression of genes in the lignin biosynthesis pathways such as *PAL*, *C4H*, *4CL*, *CCoAOMT*, *CCR*, and *CAD* genes via binding to the promoters of these genes [38,59]. Downstream of MYB46/MYB83, multiple MYB TFs (e.g., MYB4, 7, 32, 58, 63, and 85) have been shown to be specific regulators of lignin biosynthesis in *Arabidopsis* (reviewed in [40,41,42]).

BSR-Seq in the present study revealed many DEGs between the mutant and WT including seven NAC and 10 MYB TF genes (Table S7, Figure 7). Many of these DEGS are involved in the biosynthesis pathways for lignin and xylan, which were down-regulated in the mutant from a very early stage of seed development. Many of these DEGs were also identified in the transcriptomes of *C. pepo hull-less* mutant and WT [19,24,26]. Thus, our data support a similar mechanism identified in *Arabidopsis* and other plant species that NST1 is a master transcription regulator for secondary cell wall and seed coat development (Figure 7).

Recent discoveries of new transcription factors in *Arabidopsis*, and other plant species suggest that the regulatory network of lignin biosynthesis in plants may extend beyond the NAC-MYB network [41,60]. This could also be true in *C. moschata* from the data of the present study. The expression of *CmNST1* was higher in the mutant than in the WT before 20 DAP (Figure 3) whereas most genes involved in lignin biosynthesis or secondary cell wall development were down-regulated in the mutant throughout seed development (Figure 7; Table S7). Histological characterization of hull-less pumpkin seeds reveals that seed coat development is similar in both hulled and hull-less seeds in the first 10–15 days after pollination [21,25,26]. There were no significant differences in the seed coats between the mutant and WT in lignin content, and enzymatic activities for PAL, 4CL, C4H, and CAD [25]. Why does *NST1* show higher expression in the *hull-less* mutant of the present study or the *C. pepo hull-less* mutant [19] and the non-lignification mutant in *M. truncatula* [44] than in the respective wildtype plant is an interesting question. NST1 is a master TF to regulate secondary wall synthesis and its expression must be under strict developmental regulation [38,59,60]. For example, in Arabidopsis, the SND1 (for secondary wall-associated NAC domain protein) is a homolog of NST1 [55]. Wang et al. (2011) reasoned that the expression of *SND1* is under both positive regulation by its own translation product and negative regulation by downstream MYB TFs (Figure 8). Thus, these effects are presumably balanced until the balance is broken by a mutation or changing environmental factors. The elevated *NST1* transcription in the mutant may suggest that the positive autoregulation is overridden by negative regulators under normal circumstances. Fine tuning of *NST1* expression by balancing activation and repression may enable plants to adapt to ever changing environmental conditions [55]. However, other regulatory mechanisms independent of *CmNST*1 may also be present. How alternative splicing results in transcription changes and downstream targets, in particular, will be interesting subjects of future research.

## Data Availability

All data pertinent to the reported work have been provided in the manuscript or in the supplemental online materials.

## Supplementary Materials

The following supporting information can be downloaded at https://www.mdpi.com/article/10.3390/genes14050962/s1. Figure S1: Genome-wide plotting of Euclidean distance (ED) values. Figure S2: FKPM values of 14 genes in 389.5 kb region at different seed coat development stages. Figure S3: Spearman’s rank correlation coefficient (*r_s_*) plot among three replications of RNA-Seq samples. Supplemental Table S1: Information on molecular markers and primers used in this study for various purposes. Supplemental Table S2: BSA-Seq statistics. Supplemental Table S3: Annotated genes in 389.5 kb target region of the *N* locus. Supplemental Table S4: SNP allele distribution among reference region, mutant and WT bulks, and parental lines in 389.5 kb candidate gene region on Chr17. Supplemental Table S5: BSR-Seq statistics of 24 seed coat samples at four development stages from WT and mutant. Supplemental Table S6: DEGs in transcriptomes from different comparisons between the mutant and WT at four development stages. Supplemental Table S7: Differential expressed genes (DEGs) between mutant and WT seed coats at 10 days after pollination from RNA-Seq data. Supplemental File S1: Genomic and cDNA sequences of *CmNST1* in *C. moschata* and alignment of these sequences. Supplemental File S2: Genomic and mRNA sequences of *CmNST1* in *C. maxima* (Cmax), *C. pepo* (Cp), and *C. moschata* and alignment of these sequences.
