# Supplementary material for: Alternative Splicing of NAC Transcription Factor Gene CmNST1 Is Associated with Naked Seed Mutation in Pumpkin, Cucurbita moschata
Source: Genes (Basel). 2023 Apr 23;14(5):962. doi: 10.3390/genes14050962 (PMC10217548; doi:10.3390/genes14050962)
Supplement: Supplementary file 1 [file genes-14-00962-s001.zip › Fig. S1_v2.0-1.pptx]

## Slide 1
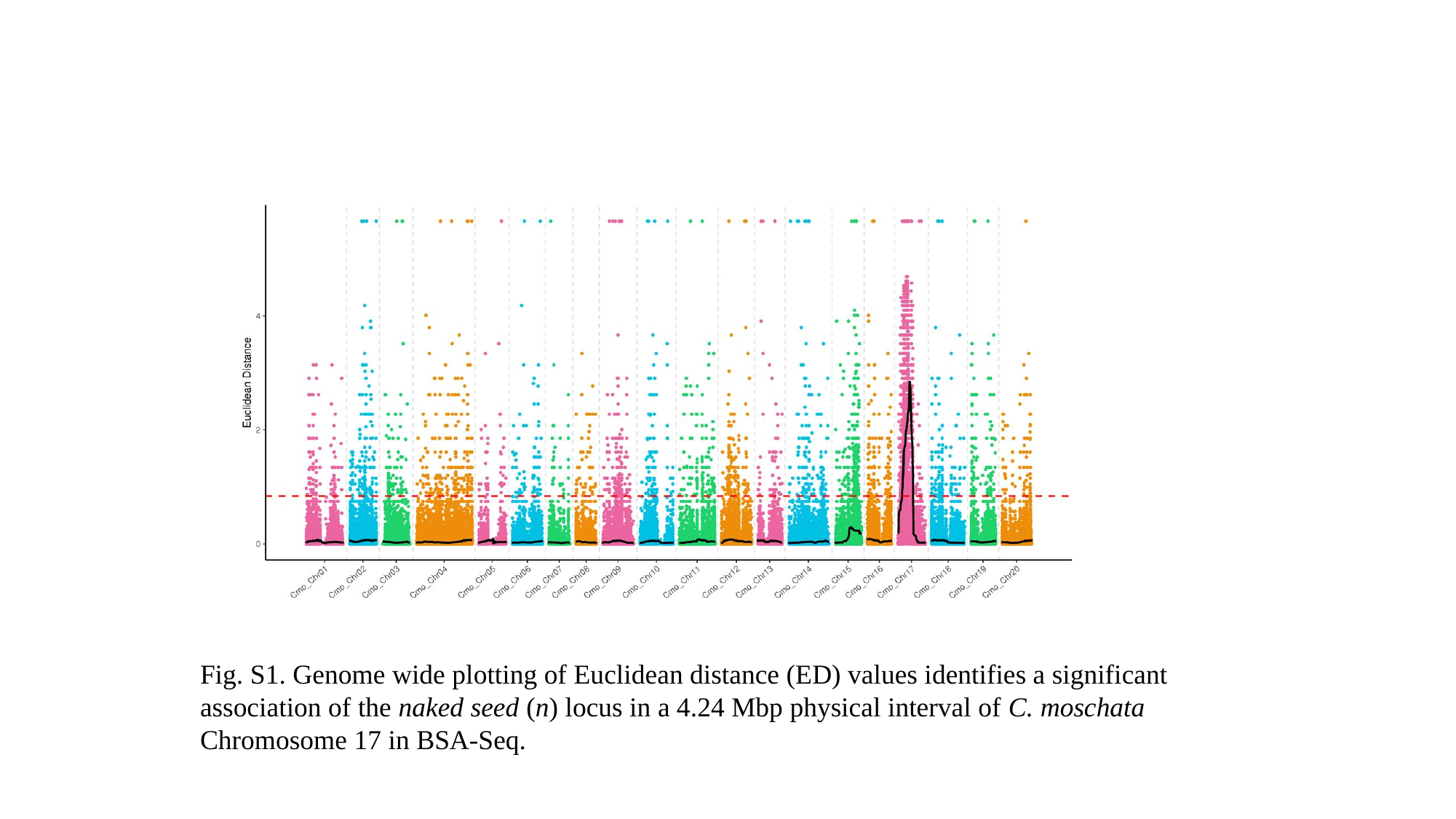

Fig. S1. Genome wide plotting of Euclidean distance (ED) values identifies a significant association of the naked seed (n) locus in a 4.24 Mbp physical interval of C. moschata Chromosome 17 in BSA-Seq.
