# Supplementary material for: Alternative Splicing of NAC Transcription Factor Gene CmNST1 Is Associated with Naked Seed Mutation in Pumpkin, Cucurbita moschata
Source: Genes (Basel). 2023 Apr 23;14(5):962. doi: 10.3390/genes14050962 (PMC10217548; doi:10.3390/genes14050962)
Supplement: Supplementary file 1 [file genes-14-00962-s001.zip › Fig. S3_v2.0-1.pptx]

## Slide 1
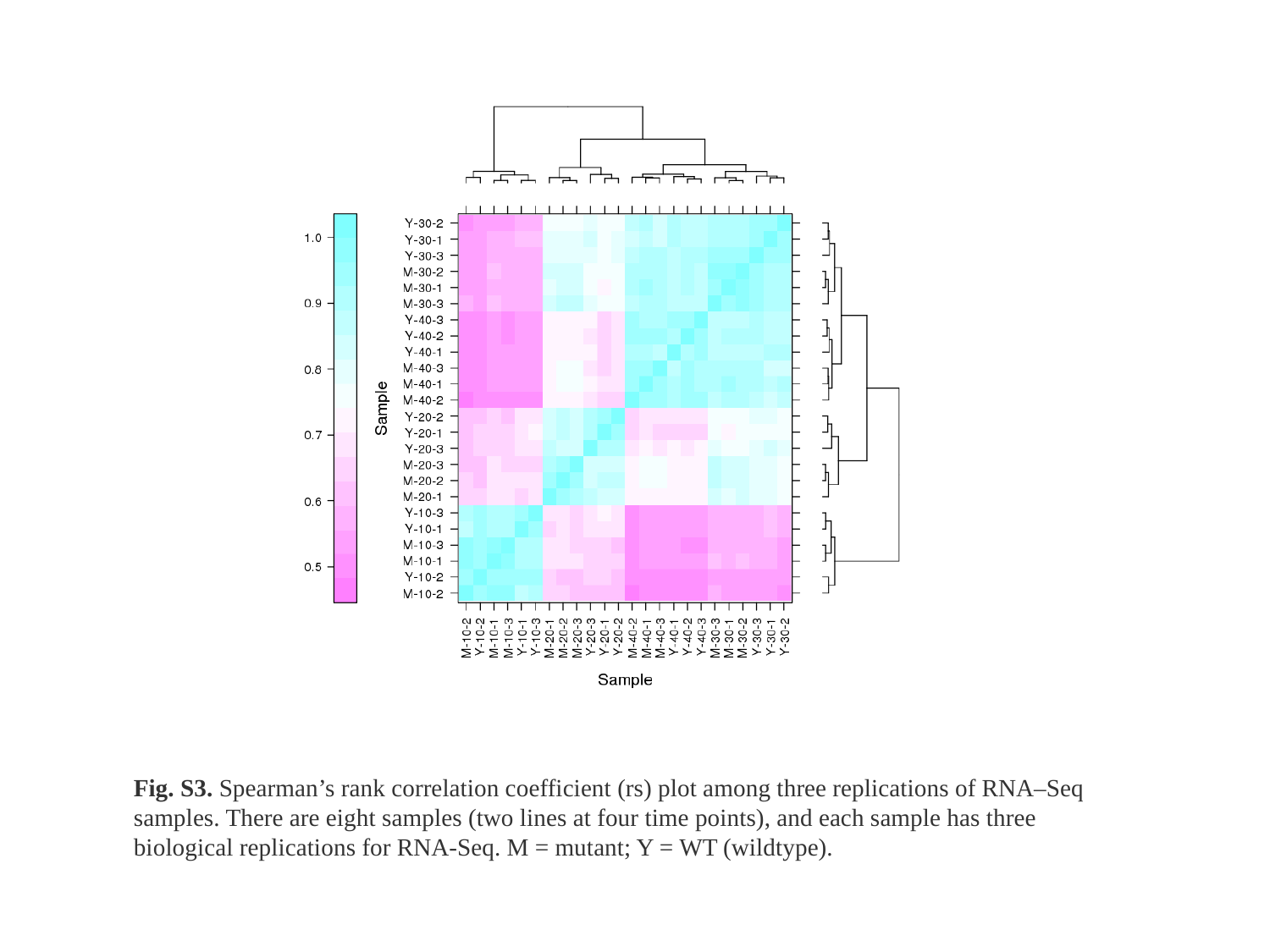

Fig. S3. Spearman’s rank correlation coefficient (rs) plot among three replications of RNA–Seq samples. There are eight samples (two lines at four time points), and each sample has three biological replications for RNA-Seq. M = mutant; Y = WT (wildtype).
