# Supplementary material for: Alternative Splicing of NAC Transcription Factor Gene CmNST1 Is Associated with Naked Seed Mutation in Pumpkin, Cucurbita moschata
Source: Genes (Basel). 2023 Apr 23;14(5):962. doi: 10.3390/genes14050962 (PMC10217548; doi:10.3390/genes14050962)
Supplement: Supplementary file 1 [file genes-14-00962-s001.zip › Supplemental File 1_final.pdf]

**Supplemental file 1.** Genomic and cDNA sequences of *CmNST1* in *C. moschata* and alignment of these sequences. M = mutant (HLS-B), WT = wildtype (HS-A), yl = young leaf, sc = seed coat, Ref = reference genome (WT)

**A. gDNA and cDNA sequence in FASTA format**

```
>WT_sc_gDNA

GCTCTTCTCCAAGCATCATACTAAAACTATACTTGCTTCAACTATATATGCCAACCCCTCTCTGTTTCCAAACAAAA
ACATAACACACACACACACACACACACACCCAAATGAGTATCTCCGTGAATGGCCACTCCCAAGTCCCTCCCGGCTT
CCGCTTCCACCCCACCGAAGAGGAGCTCCTCCACTACTACCTCCGCAAGAAGCTCTCCTTCCACACCGTCGATTTGG
ATGTCATTCCCGATGTCGACCTCAACAAGCTCGAGCCGTGGGACATCCAAGGTACGTATCTCATTTTCTCCTCTCAT
TTTTATTGCCTTTCCTCCTCTCATTCAATTACACTTTCTAGAGAAATGCAAGATCGGAACCACTCCTCAAAACGATT
GGTACTTTTTTAGTCATAAAGATAAAAAGTATCCCACCGGTACTCGCACCAATCGCGCCACCGCTGCTGGCTTCTGG
AAGGCCACGGGTCGTGATAAAGTCATTTACACCAACTCCCGCCGAATTGGTATGAGAAAGACTCTTGTTTTTTATAA
AGGTCGCGCCCCTCACGGCCAGAAGTCTGATTGGATCATGCATGAATATCGCCTCGATGAAATTTCCACCTCCCAAT
CCAGTAATGTATGTTTGTTTGTCTTATCTCGTTAAGAATTAGAAACATAAGTGGGATTTTCTTGGATTCCAGTTATG
TTTTCCATTCTTAACATGGTATCAGATTAGAGGTTGTTATTGTTAATGTATCTCTAAATGGTTCGGTTTGATTTAGA
TTAAGGCGTCGTCGAGCGGTGTGATTGGAGACGGAGGACAAGAAGAGGGGTGGGTTGTGTGTAGGATATTCAAGAAG
AAAAACCACCACAAAACCCTGGACAGCCCTGTCAGTACTACAATTACCGAAACACCCTCCTCTCTCTTGCTTGATTC
TTGCAATGACGGAGGCGTTGGAGCAAATCTTTCATTACATGGGTCGAAGCTGCAAGGACATAGAAGACGGAGACGGA
GACGGAGACGGAGACGGCGGCGGTGGGAGATTGCTCAGCCCCATCGACACTTCCACTGGCGGTAGTTACCTGGATGG
CAGATTCTCCAAACTTCCAAACCTCGAAAGCCCCAATTCCACCAGCACCCACAACTGCCACCAACCCATTAACAACC
ATGTGGGCCCCACCGATCCGATTCTAATTTCCGGCTACCAACTGGAGTCCTCTTCTGCCGCCGCGGCTACAGCTCCT
CACAACTGGGCGGCGTTCGACCGGCTGGTGGCCTCGCAACTGAACGGCCAAATTGAAGTGTCCAATATGATTTATTA
CAATGACCAATTACCAACTCCACCAACACTACGCGGAACGACATCGTTTTCGTCCAAATCATCCTCCTCTTCTTACA
CCGCCGCCGCCGGTCAGGATTACAACAACGTGGACACAGAGCTGTGGAGCTTTGCGAAGTTGTCGTCATCCCTGTCG
TCGTCGTCTGACCCATTATGCCACGTGTCCAACACTCCAATATAGCTGCTACTGAGAATCATCCACGCGAAGAGAGA
GAAAGAGAGAAGAATTTGAGAAACAAGGTTT

>WT_sc_cDNA

TTTTCTCACCCTTCCTTGCTCTTCTCCAAGCATCATACTAAAACTATACTTGCTTCAACTATATATGCCAACCCCTC
TCTGTTTCCAAACAAAAACATAACACACACACACACACACACACACCCAAATGAGTATCTCCGTGAATGGCCACTCC
CAAGTCCCTCCCGGCTTCCGCTTCCACCCCACCGAAGAGGAGCTCCTCCACTACTACCTCCGCAAGAAGCTCTCCTT
CCACACCGTCGATTTGGATGTCATTCCCGATGTCGACCTCAACAAGCTCGAGCCGTGGGACATCCAAGAGAAATGCA
AGATCGGAACCACTCCTCAAAACGATTGGTACTTTTTTAGTCATAAAGATAAAAAGTATCCCACCGGTACTCGCACC
AATCGCGCCACCGCTGCTGGCTTCTGGAAGGCCACGGGTCGTGATAAAGTCATTTACACCAACTCCCGCCGAATTGG
TATGAGAAAGACTCTTGTTTTTTATAAAGGTCGCGCCCCTCACGGCCAGAAGTCTGATTGGATCATGCATGAATATC
GCCTCGATGAAATTTCCACCTCCCAATCCAGTAATATTAAGGCGTCGTCGAGCGGTGTGATTGGAGACGGAGGACAA
GAAGAGGGGTGGGTTGTGTGTAGGATATTCAAGAAGAAAAACCACCACAAAACCCTGGACAGCCCTGTCAGTACTAC
AATTACCGAAACACCCTCCTCTCTCTTGCTTGATTCTTGCAATGACGGAGCGTTGGAGCAAATCTTTCATTACATGG
GTCGAAGCTGCAAGGACATAGAAGACGGAGACGGAGACGGAGACGGAGACGGCGGCGGTGGGAGATTGCTCAGCCCC
ATCGACACTTCCACTGGCGGTAGTTACCTGGATGGCAGATTCTCCAAACTTCCAAACCTCGAAAGCCCCAATTCCAC
CAGCACCCACAACTGCCACCAACCCATTAACAACCATGTGGGCCCCACCGATCCGATTCTAATTTCCGGCTACCAAC
TGGAGTCCTCTTCTGCCGCCGCGGCTACAGCTCCTCACAACTGGGCGGCGTTCGACCGGCTGGTGGCCTCGCAACTG
AACGGCCAAATTGAAGTGTCCAATATGATTTATTACAATGACCAATTACCAACTCCACCAACACTACGCGGAACGAC
ATCGTTTTCGTCCAAATCATCCTCCTCTTCTTACACCGCCGCCGCCGGTCAGGATTACAACAACGTGGACACAGAGC
TGTGGAGCTTTGCGAAGTTGTCGTCATCCCTGTCGTCGTCGTCTGACCCATTATGCCACGTGTCCAACACTCCAATA
TAGCTGCTACTGAGAATCATCCACGCCGAAG

>M_sc_gDNA
```

GCTCTTCTCCAAGCATCATACTAAAACTATACTTGCTTCAACTATATATGCCAACCCCTCTCTGTTTCCAAACAAAA
ACATAACACACACACACACACACACACACCCAAATGAGTATCTCCGTGAATGGCCACTCCCAAGTCCCTCCCGGCTT
CGCTTCCACCCCACCGAAGAGGAGCTCCTCCACTACTACCTCCGCAAGAAGCTCTCCTTCCACACCGTCGATTTGGA
TGTCATTCCCGATGTCGACCTCAACAAGCTCGAGCCGTGGGACATCCAAGGTACGTATCTCATTTTCTCCTCTCATT
TTTATTGCCTTTCCTCCTCTCATTCAATTACACTTTCTAGAGAAATGCAAGATCGGAACCACTCCTCAAAACGATTG
GTACTTTTTTAGTCATAAAGATAAAAAGTATCCCACCGGTACTCGCACCAATCGCGCCACCGCTGCTGGCTTCTGGA
AGGCCACGGGTCGTGATAAAGTCATTTACACCAACTCCCGCCGAATTGGTATGAGAAAGACTCTTGTTTTTTATAAA
GGTCGCGCCCCTCACGGCCAGAAGTCTGATTGGATCATGCATGAATATCGCCTCGATGAAATTTCCACCTCCCAATC
CAGTAATGTATGTTTGTTTGTCTTATCTCGTTAAGAATTAGAAACATAAGTGGGATTTTCTTGGATTCCAGTTATGT
TTTCCATTCTTAACATGGTATCAGATTAGAGGTTGTTATTGTTAATGTATCTCTAAATGGTTCGGTTTGATTTAGAT
TAAGGCGTCGTCGAGCGGTGTGATTGGAGACGGAGGACAAGAAGAGGGGTGGGTTGTGTGTAGGATATTCAAGAAGA
AAAACCACCACAAAACCCTGGACAGCCCTGTCAGTACTACAATTACCGAAACACCCTCCTCTCTCTTGCTTGATTCT
TGCAATGACGGAGGCGTTGGAGCAAATCTTTCATTACATGGGTCGAAGCTGCAAGGACATAGAAGACGGAGACGGAG
ACGGAGACGGAGACGGCGGCGGTGGGAGATTGCTCAGCCCCATCGACACTTCCACTGGCGGTAGTTACCTGGATGGC
AGATTCTCCAAACTTCCAAACCTCGAAAGCCCCAATTCCACCAGCACCCACAACTGCCACCAACCCATTAACAACCA
TGTGGGCCCCACCGATCCGATTCTAATTTCCGGCTACCAACTGGAGTCCTCTTCTGCCGCCGCGGCTACAGCTCCTC
ACAACTGGGCGGCGTTCGACCGGCTGGTGGCCTCGCAACTGAACGGCCAAATTGAAGTGTCCAATATGATTTATTAC
AATGACCAATTACCAACTCCACCAACACTACGCGGAACGACATCGTTTTCGTCCAAATCATCCTCCTCTTCTTACAC
CGCCGCCGCCGGTCAGGATTACAACAACGTGGACACAGAGCTGTGGAGCTTTGCGAAGTTGTCGTCATCCCTGTCGT
CGTCGTCTGACCCATTATGCCACGTGTCCAACACTCCAATATAGCTGCTACTGAGAATCATCCACGCGAAGAGAGAG
AAAGAGAGAAGAATTTGAGAAACCAAGGTTT

>M_sc_cDNA

TTGCTCTTCTCCAAGCATCATACTAAAACTATACTTGCTTCAACTATATATGCCAACCCCTCTCTGTTTCCAAACAA
AAACATAACACACACACACACACACACACACCCAAATGAGTATCTCCGTGAATGGCCACTCCCAAGTCCCTCCCGGC
TTCCGCTTCCACCCCACCGAAGAGGAGCTCCTCCACTACTACCTCCGCAAGAAGCTCTCCTTCCACACCGTCGATTT
GGATGTCATTCCCGATGTCGACCTCAACAAGCTCGAGCCGTGGGACATCCAAGAGAAATGCAAGATCGGAACCACTC
CTCAAAACGATTGGTACTTTTTTAGTCATAAAGATAAAAAGTATCCCACCGGTACTCGCACCAATCGCGCCACCGCT
GCTGGCTTCTGGAAGGCCACGGGTCGTGATAAAGTCATTTACACCAACTCCCGCCGAATTGATTAAGGCGTCGTCGA
GCGGTGTGATTGGAGACGGAGGACAAGAAGAGGGGTGGGTTGTGTGTAGGATATTCAAGAAGAAAAACCACCACAAA
ACCCTGGACAGCCCTGTCAGTACTACAATTACCGAAACACCCTCCTCTCTCTTGCTTGATTCTTGCAATGACGGAGC
GTTGGAGCAAATCTTTCATTACATGGGTCGAAGCTGCAAGGACATAGAAGACGGAGACGGAGACGGAGACGGAGACG
GCGGCGGTGGGAGATTGCTCAGCCCCATCGACACTTCCACTGGCGGTAGTTACCTGGATGGCAGATTCTCCAAACTT
CCAAACCTCGAAAGCCCCAATTCCACCAGCACCCACAACTGCCACCAACCCATTAACAACCATGTGGGCCCCACCGA
TCCGATTCTAATTTCCGGCTACCAACTGGAGTCCTCTTCTGCCGCCGCGGCTACAGCTCCTCACAACTGGGCGGCGT
TCGACCGGCTGGTGGCCTCGCAACTGAACGGCCAAATTGAAGTGTCCAATATGATTTATTACAATGACCAATTACCA
ACTCCACCAACACTACGCGGAACGACATCGTTTTCGTCCAAATCATCCTCCTCTTCTTACACCGCCGCCGCCGGTCA
GGATTACAACAACGTGGACACAGAGCTGTGGAGCTTTGCGAAGTTGTCGTCATCCCTGTCGTCGTCGTCTGACCCAT
TATGCCACGTGTCCAACACTCCAATATAGCTGCTACTGAGAATCATCCACGC

>M-yl_cDNA

TATGGGACCCATTTCCGTGTTGTAATCATATTCTCACACCCTTCCTTGCTCTTCTCCAAGCATCATACTAAAACTAC
ACTTGCTTCAACTATATATGCCAACCCCTCTCTGTTTCCAAACAAAAACATAACACACACACACACACACACACACC
CAAATGAGTATCTCCGTGAATGGCCACTCCCAAGTCCCTCCCGGCTTCGCTTCCACCCCACCGAAGAGGAGCTCCTC
CACTACTACCTCCGCAAGAAGCTCTCCTTCCACACCGTCGATTTGGATGTCATTCCCGATGTCGACCTCAACAAGCT
CGAGCCGTGGGACATCCAAGAGAAATGCAAGATCGGAACCACTCCTCAAAACGATTGGTACTTTTTTAGTCATAAAG
ATAAAAAGTATCCCACCGGTACTCGCACCAATCGCGCCACCGCTGCTGGCTTCTGGAAGGCCACGGGTCGTGATAAA
GTCATTTACACCAACTCCCGCCGAATTGGTATGAGAAAGACTCTTGTTTTTTATAAAGGTCGCGCCCCTCACGGCCA
GAAGTCTGATTGGATCATGCATGAATATCGCCTCGATGAAATTTCCACCTCCCAATCCAGTAATATTAAGGCGTCGT
CGAGCGGTGTGATTGGAGACGGAGGACAAGAAGAGGGGTGGGTTGTGTGTAGGATATTCAAGAAGAAAAACCACCAC
AAAACCCTGGACAGCCCTGTCAGTACTACAATTACCGAAACACCCTCCTCTCTCTTGCTTGATTCTTGCAATGACGG
AGGCGTTGGAGCAAATCTTTCATTACATGGGTCGAAGCTGCAAGGACATAGAAGACGGAGACGGAGACGGAGACGGA
GACGGCGGCGGTGGGAGATTGCTCAGCCCCATCGACACTTCCACTGGCGGTAGTTACCTGGATGGCAGATTCTCCAA
ACTTCCAAACCTCGAAAGCCCCAATTCCACCAGCACCCACAACTGCCACCAACCCATTAACAACCATGTGGGCCCCA

CCGATCCGATTCTAATTTCCGGCTACCAACTGGAGTCCTCTTCTGCCGCCGCGGCTACAGCTCCTCACAACTGGGCG
GCGTTCGACCGGCTGGTGGCCTCGCAACTGAACGGCCAAATTGAAGTGTCCAATATGATTTATTACAATGACCAATT
ACCAACTCCACCAACACTACGCGGAACGACATCGTTTTCGTCCAAATCATCCTCCTCTTCTTACACCGCCGCCGCCG
GTCAGGATTACAACAACGTGGACACAGAGCTGTGGAGCTTTGCGAAGTTGTCGTCATCCCTGTCGTCGTCGTCTGAC
CCATTATGCCACGTGTCCAACACTCCAATATAGCTGCTACTGAGAATCATCCACGCGAAGAGAGAGAAAGAGAGAAG
AATTTGAAGAACAAGGTT

>WT_yl_cDNA

TATGGGACCCATTTCCGTGTTGTAATCATATTCTCACACCCTTCCTTGCTCTTCTCCAAGCATCATACTAAAACTAC
ACTTGCTTCAACTATATATGCCAACCCCTCTCTGTTTCCAAACAAAAACATAACACACACACACACACACACACACC
CAAATGAGTATCTCCGTGAATGGCCACTCCCAAGTCCCTCCCGGCTTCGCTTCCACCCCACCGAAGAGGAGCTCCTC
CACTACTACCTCCGCAAGAAGCTCTCCTTCCACACCGTCGATTTGGATGTCATTCCCGATGTCGACCTCAACAAGCT
CGAGCCGTGGGACATCCAAGAGAAATGCAAGATCGGAACCACTCCTCAAAACGATTGGTACTTTTTTAGTCATAAAG
ATAAAAAGTATCCCACCGGTACTCGCACCAATCGCGCCACCGCTGCTGGCTTCTGGAAGGCCACGGGTCGTGATAAA
GTCATTTACACCAACTCCCGCCGAATTGGTATGAGAAAGACTCTTGTTTTTTATAAAGGTCGCGCCCCTCACGGCCA
GAAGTCTGATTGGATCATGCATGAATATCGCCTCGATGAAATTTCCACCTCCCAATCCAGTAATATTAAGGCGTCGT
CGAGCGGTGTGATTGGAGACGGAGGACAAGAAGAGGGGTGGGTTGTGTGTAGGATATTCAAGAAGAAAAACCACCAC
AAAACCCTGGACAGCCCTGTCAGTACTACAATTACCGAAACACCCTCCTCTCTCTTGCTTGATTCTTGCAATGACGG
AGGCGTTGGAGCAAATCTTTCATTACATGGGTCGAAGCTGCAAGGACATAGAAGACGGAGACGGAGACGGAGACGGA
GACGGCGGCGGTGGGAGATTGCTCAGCCCCATCGACACTTCCACTGGCGGTAGTTACCTGGATGGCAGATTCTCCAA
ACTTCCAAACCTCGAAAGCCCCAATTCCACCAGCACCCACAACTGCCACCAACCCATTAACAACCATGTGGGCCCCA
CCGATCCGATTCTAATTTCCGGCTACCAACTGGAGTCCTCTTCTGCCGCCGCGGCTACAGCTCCTCACAACTGGGCG
GCGTTCGACCGGCTGGTGGCCTCGCAACTGAACGGCCAAATTGAAGTGTCCAATATGATTTATTACAATGACCAATT
ACCAACTCCACCAACACTACGCGGAACGACATCGTTTTCGTCCAAATCATCCTCCTCTTCTTACACCGCCGCCGCCG
GTCAGGATTACAACAACGTGGACACAGAGCTGTGGAGCTTTGCGAAGTTGTCGTCATCCCTGTCGTCGTCGTCTGAC
CCATTATGCCACGTGTCCAACACTCCAATATAGCTGCTACTGAGAATCATCCACGCGAAGAGAGAGAAAGAGAGAAG
AATTTGAAGAACAAGGTT

>Ref_DNA

TATGGGACCCATTTCCGTGTTGTAATCATATTCTCACACCCTTCCTTGCTCTTCTCCAAGCATCATACTAAAACTAC
ACTTGCTTCAACTATATATGCCAACCCCTCTCTGTTTCCAAACAAAAACATAACACACACCCAAATGAGTATCTCCG
TGAATGGCCACTCCCAAGTCCCTCCCGGCTTCCGCTTCCACCCCACCGAAGAGGAGCTCCTCCACTACTACCTCCGC
AAGAAGCTCTCCTTCCACACCGTCGATTTGGATGTCATTCCCGATGTCGACCTCAACAAGCTCGAGCCGTGGGACAT
CCAAGGTACGTATCTCATTTTCTCCTCTCATTTTTATTGCCTTTCCTCCTCTCATTCAATTACACTTTCTAGAGAAA
TGCAAGATCGGAACCACTCCCCAAAACGATTGGTACTTTTTTAGTCATAAAGATAAAAAGTATCCCACCGGTACTCG
CACCAATCGCGCCACCGCTGCTGGCTTCTGGAAGGCCACGGGTCGTGATAAAGTCATTTACACCAACTCCCGCCGAA
TTGGTATGAGAAAGACTCTTGTTTTTTATAAAGGTCGCGCCCCTCACGGCCAGAAGTCTGATTGGATCATGCATGAA
TATCGCCTCGATGAAATTTCCACCTCCCAATCCAGTAATGTATGTCTGTTTGTCTTATCTCGTTAAGAATTAGAAAC
ATAAGTGGGATTTTCTTGGATTCCAGTTCTGTTTTCCATTCTTAACATGGTATCAGATTAGAGGTTGTTATTGTTAA
TGTATCTCTAAATGGTTCGGTTTGATTTAGATTAAGGCGTCGTCGAGCGGTGTGATTGGAGACGGAGGACAAGAAGA
GGGGTGGGTTGTGTGTAGGATATTCAAGAAGAAAAACCACCACAAAACCCTGGACAGCCCTGTCAGTACTACAATTA
CCGAAACACCCTCCTCTCTCTTGCTTGATTCTTGCAATGACGGAGCGTTGGAGCAAATCTTTCATTACATGGGTCGA
AGCTGCAAGGACATAGAAGACGGAGACGGAGACGGAGACGGAGACGGCGGCGGTGGGAGATTGCTCAGCCCCATCGA
CACTTCCACTGGCGGTAGTTACCTGGATGGCAGATTCTCCAAACTTCCAAACCTCGAAAGCCCCAATTCCACCAGCA
CCCACAACTGCCACCAACCCATTAACAACCATGTGGGCCCCACCGATCCGATTCTAATTTCCGGCTACCAACTGGAG
TCCTCTTCTGCCGCCGCGGCTACAGCTCCTCACAACTGGGCGGCGTTCGACCGGCTGGTGGCCTCGCAACTGAACGG
CCAAATTGAAGTGTCCAATATGATTTATTACAATGACCAATTACCAACTCCACCAACACTACGCGGAACGACATCGT
TTTCGTCCAAATCATCCTCCTCTTCTTACACCGCCGCCGCCGGTCAGGATTACAACAACGTGGACACAGAGCTGTGG
AGCTTTGCGAAGTTGTCGTCATCCCTGTCGTCGTCGTCTGACCCATTATGCCACGTGTCCAACACTCCAATATAGCT
GCTACTGAGAATCATCCACGCGAAGAGAGAGAAAGAGAGAAGAATTTGAAGACAAGGTTTTTAATTAATTAATTAAA
TATTTACGTATGTGATGAA

>Ref_mRNA

TATGGGACCCATTTCCGTGTTGTAATCATATTCTCACACCCTTCCTTGCTCTTCTCCAAGCATCATACTAAAACTAC
ACTTGCTTCAACTATATATGCCAACCCCTCTCTGTTTCCAAACAAAAACATAACACACACCCAAATGAGTATCTCCG
TGAATGGCCACTCCCAAGTCCCTCCCGGCTTCCGCTTCCACCCCACCGAAGAGGAGCTCCTCCACTACTACCTCCGC
AAGAAGCTCTCCTTCCACACCGTCGATTTGGATGTCATTCCCGATGTCGACCTCAACAAGCTCGAGCCGTGGGACAT
CCAAGAGAAATGCAAGATCGGAACCACTCCCCAAAACGATTGGTACTTTTTTAGTCATAAAGATAAAAAGTATCCCA
CCGGTACTCGCACCAATCGCGCCACCGCTGCTGGCTTCTGGAAGGCCACGGGTCGTGATAAAGTCATTTACACCAAC
TCCCGCCGAATTGGTATGAGAAAGACTCTTGTTTTTTATAAAGGTCGCGCCCCTCACGGCCAGAAGTCTGATTGGAT
CATGCATGAATATCGCCTCGATGAAATTTCCACCTCCCAATCCAGTAATATTAAGGCGTCGTCGAGCGGTGTGATTG
GAGACGGAGGACAAGAAGAGGGGTGGGTTGTGTGTAGGATATTCAAGAAGAAAAACCACCACAAAACCCTGGACAGC
CCTGTCAGTACTACAATTACCGAAACACCCTCCTCTCTCTTGCTTGATTCTTGCAATGACGGAGCGTTGGAGCAAAT
CTTTCATTACATGGGTCGAAGCTGCAAGGACATAGAAGACGGAGACGGAGACGGAGACGGAGACGGCGGCGGTGGGA
GATTGCTCAGCCCCATCGACACTTCCACTGGCGGTAGTTACCTGGATGGCAGATTCTCCAAACTTCCAAACCTCGAA
AGCCCCAATTCCACCAGCACCCACAACTGCCACCAACCCATTAACAACCATGTGGGCCCCACCGATCCGATTCTAAT
TTCCGGCTACCAACTGGAGTCCTCTTCTGCCGCCGCGGCTACAGCTCCTCACAACTGGGCGGCGTTCGACCGGCTGG
TGGCCTCGCAACTGAACGGCCAAATTGAAGTGTCCAATATGATTTATTACAATGACCAATTACCAACTCCACCAACA
CTACGCGGAACGACATCGTTTTCGTCCAAATCATCCTCCTCTTCTTACACCGCCGCCGCCGGTCAGGATTACAACAA
CGTGGACACAGAGCTGTGGAGCTTTGCGAAGTTGTCGTCATCCCTGTCGTCGTCGTCTGACCCATTATGCCACGTGT
CCAACACTCCAATATAGCTGCTACTGAGAATCATCCACGCGAAGAGAGAGAAAGAGAGAAGAATTTGAAGACAAGGT
TTTTAATTAATTAATTAAATATTTACGTATGTGATGAA

**B. Alignment of cDNA and gDNA sequences. M = mutant (HLS-B), WT = wildtype (HS-A), yl = young leaf, sc = seed coat, Ref = reference genome (WT)**

```
M-yl_cDNA       TATGGGACCCATTTCCGTGTTGTAATCATATTCTCACACCCTTCCTTGCTCTTCTCCAAG 60
WT_yl_cDNA      TATGGGACCCATTTCCGTGTTGTAATCATATTCTCACACCCTTCCTTGCTCTTCTCCAAG 60
WT_sc_cDNA      ------------------------------TTTTCTCACCCTTCCTTGCTCTTCTCCAAG 30
M_sc_cDNA       ---------------------------------------------TTGCTCTTCTCCAAG 15
WT_sc_gDNA      -----------------------------------------------GCTCTTCTCCAAG 13
M_sc_gDNA       -----------------------------------------------GCTCTTCTCCAAG 13
Ref_DNA         TATGGGACCCATTTCCGTGTTGTAATCATATTCTCACACCCTTCCTTGCTCTTCTCCAAG 60
Ref_mRNA        TATGGGACCCATTTCCGTGTTGTAATCATATTCTCACACCCTTCCTTGCTCTTCTCCAAG 60
                                                               *************

M-yl_cDNA       CATCATACTAAAACTACACTTGCTTCAACTATATATGCCAACCCCTCTCTGTTTCCAAAC 120
WT_yl_cDNA      CATCATACTAAAACTACACTTGCTTCAACTATATATGCCAACCCCTCTCTGTTTCCAAAC 120
WT_sc_cDNA      CATCATACTAAAACTATACTTGCTTCAACTATATATGCCAACCCCTCTCTGTTTCCAAAC 90
M_sc_cDNA       CATCATACTAAAACTATACTTGCTTCAACTATATATGCCAACCCCTCTCTGTTTCCAAAC 75
WT_sc_gDNA      CATCATACTAAAACTATACTTGCTTCAACTATATATGCCAACCCCTCTCTGTTTCCAAAC 73
M_sc_gDNA       CATCATACTAAAACTATACTTGCTTCAACTATATATGCCAACCCCTCTCTGTTTCCAAAC 73
Ref_DNA         CATCATACTAAAACTACACTTGCTTCAACTATATATGCCAACCCCTCTCTGTTTCCAAAC 120
Ref_mRNA        CATCATACTAAAACTACACTTGCTTCAACTATATATGCCAACCCCTCTCTGTTTCCAAAC 120
                **************** *******************************************

M-yl_cDNA       AAAAACATAACACACACACACACACACACACACACCCAAATGAGTATCTCCGTGAATGGCCA 180
WT_yl_cDNA      AAAAACATAACACACACACACACACACACACACACCCAAATGAGTATCTCCGTGAATGGCCA 180
WT_sc_cDNA      AAAAACATAACACACACACACACACACACACACACCCAAATGAGTATCTCCGTGAATGGCCA 150
M_sc_cDNA       AAAAACATAACACACACACACACACACACACACACCCAAATGAGTATCTCCGTGAATGGCCA 135
WT_sc_gDNA      AAAAACATAACACACACACACACACACACACACACCCAAATGAGTATCTCCGTGAATGGCCA 133
M_sc_gDNA       AAAAACATAACACACACACACACACACACACACACCCAAATGAGTATCTCCGTGAATGGCCA 133
Ref_DNA         AAAAACATAACA----------------CACACCCAAATGAGTATCTCCGTGAATGGCCA 164
Ref_mRNA        AAAAACATAACA----------------CACACCCAAATGAGTATCTCCGTGAATGGCCA 164
                ************                ********************************
                             SSR                        TSS & Exon 1 start

M-yl_cDNA       CTCCCAAGTCCCTCCCGGCTTC-GCTTCCACCCCACCGAAGAGGAGCTCCTCCACTACTA 239
WT_yl_cDNA      CTCCCAAGTCCCTCCCGGCTTC-GCTTCCACCCCACCGAAGAGGAGCTCCTCCACTACTA 239
WT_sc_cDNA      CTCCCAAGTCCCTCCCGGCTTCCGCTTCCACCCCACCGAAGAGGAGCTCCTCCACTACTA 210
M_sc_cDNA       CTCCCAAGTCCCTCCCGGCTTCCGCTTCCACCCCACCGAAGAGGAGCTCCTCCACTACTA 195
WT_sc_gDNA      CTCCCAAGTCCCTCCCGGCTTCCGCTTCCACCCCACCGAAGAGGAGCTCCTCCACTACTA 193
M_sc_gDNA       CTCCCAAGTCCCTCCCGGCTTC-GCTTCCACCCCACCGAAGAGGAGCTCCTCCACTACTA 192
Ref_DNA         CTCCCAAGTCCCTCCCGGCTTCCGCTTCCACCCCACCGAAGAGGAGCTCCTCCACTACTA 224
Ref_mRNA        CTCCCAAGTCCCTCCCGGCTTCCGCTTCCACCCCACCGAAGAGGAGCTCCTCCACTACTA 224
                ********************** *************************************

M-yl_cDNA       CCTCCGCAAGAAGCTCTCCTTCCACACCGTCGATTTGGATGTCATTCCCGATGTCGACCT 299
WT_yl_cDNA      CCTCCGCAAGAAGCTCTCCTTCCACACCGTCGATTTGGATGTCATTCCCGATGTCGACCT 299
WT_sc_cDNA      CCTCCGCAAGAAGCTCTCCTTCCACACCGTCGATTTGGATGTCATTCCCGATGTCGACCT 270
M_sc_cDNA       CCTCCGCAAGAAGCTCTCCTTCCACACCGTCGATTTGGATGTCATTCCCGATGTCGACCT 255
WT_sc_gDNA      CCTCCGCAAGAAGCTCTCCTTCCACACCGTCGATTTGGATGTCATTCCCGATGTCGACCT 253
M_sc_gDNA       CCTCCGCAAGAAGCTCTCCTTCCACACCGTCGATTTGGATGTCATTCCCGATGTCGACCT 252
Ref_DNA         CCTCCGCAAGAAGCTCTCCTTCCACACCGTCGATTTGGATGTCATTCCCGATGTCGACCT 284
Ref_mRNA        CCTCCGCAAGAAGCTCTCCTTCCACACCGTCGATTTGGATGTCATTCCCGATGTCGACCT 284
                ************************************************************

M-yl_cDNA       CAACAAGCTCGAGCCGTGGGACATCCAA-------------------------------- 327
WT_yl_cDNA      CAACAAGCTCGAGCCGTGGGACATCCAA-------------------------------- 327
WT_sc_cDNA      CAACAAGCTCGAGCCGTGGGACATCCAA-------------------------------- 298
M_sc_cDNA       CAACAAGCTCGAGCCGTGGGACATCCAA-------------------------------- 283
WT_sc_gDNA      CAACAAGCTCGAGCCGTGGGACATCCAAGGTACGTATCTCATTTTCTCCTCTCATTTTTA 313
M_sc_gDNA       CAACAAGCTCGAGCCGTGGGACATCCAAGGTACGTATCTCATTTTCTCCTCTCATTTTTA 312
Ref_DNA         CAACAAGCTCGAGCCGTGGGACATCCAAGGTACGTATCTCATTTTCTCCTCTCATTTTTA 344
```

```
Ref_mRNA        CAACAAGCTCGAGCCGTGGGACATCCA--------------------------------- 311
                ***************************
                                            Intron 1 start


M-yl_cDNA       -----------------------------------GAGAAATGCAAGATCGGAACCACTC 352
WT_yl_cDNA      -----------------------------------GAGAAATGCAAGATCGGAACCACTC 352
WT_sc_cDNA      -----------------------------------GAGAAATGCAAGATCGGAACCACTC 323
M_sc_cDNA       -----------------------------------GAGAAATGCAAGATCGGAACCACTC 308
WT_sc_gDNA      TTGCCTTTCCTCCTCTCATTCAATTACACTTTCTAGAGAAATGCAAGATCGGAACCACTC 373
M_sc_gDNA       TTGCCTTTCCTCCTCTCATTCAATTACACTTTCTAGAGAAATGCAAGATCGGAACCACTC 372
Ref_DNA         TTGCCTTTCCTCCTCTCATTCAATTACACTTTCTAGAGAAATGCAAGATCGGAACCACTC 404
Ref_mRNA        ----------------------------------AGAGAAATGCAAGATCGGAACCACTC 337
                                                   *************************

M-yl_cDNA       CTCAAAACGATTGGTACTTTTTTAGTCATAAAGATAAAAAGTATCCCACCGGTACTCGCA 412
WT_yl_cDNA      CTCAAAACGATTGGTACTTTTTTAGTCATAAAGATAAAAAGTATCCCACCGGTACTCGCA 412
WT_sc_cDNA      CTCAAAACGATTGGTACTTTTTTAGTCATAAAGATAAAAAGTATCCCACCGGTACTCGCA 383
M_sc_cDNA       CTCAAAACGATTGGTACTTTTTTAGTCATAAAGATAAAAAGTATCCCACCGGTACTCGCA 368
WT_sc_gDNA      CTCAAAACGATTGGTACTTTTTTAGTCATAAAGATAAAAAGTATCCCACCGGTACTCGCA 433
M_sc_gDNA       CTCAAAACGATTGGTACTTTTTTAGTCATAAAGATAAAAAGTATCCCACCGGTACTCGCA 432
Ref_DNA         CCCAAAACGATTGGTACTTTTTTAGTCATAAAGATAAAAAGTATCCCACCGGTACTCGCA 464
Ref_mRNA        CCCAAAACGATTGGTACTTTTTTAGTCATAAAGATAAAAAGTATCCCACCGGTACTCGCA 397
                * **********************************************************


M-yl_cDNA       CCAATCGCGCCACCGCTGCTGGCTTCTGGAAGGCCACGGGTCGTGATAAAGTCATTTACA 472
WT_yl_cDNA      CCAATCGCGCCACCGCTGCTGGCTTCTGGAAGGCCACGGGTCGTGATAAAGTCATTTACA 472
WT_sc_cDNA      CCAATCGCGCCACCGCTGCTGGCTTCTGGAAGGCCACGGGTCGTGATAAAGTCATTTACA 443
M_sc_cDNA       CCAATCGCGCCACCGCTGCTGGCTTCTGGAAGGCCACGGGTCGTGATAAAGTCATTTACA 428
WT_sc_gDNA      CCAATCGCGCCACCGCTGCTGGCTTCTGGAAGGCCACGGGTCGTGATAAAGTCATTTACA 493
M_sc_gDNA       CCAATCGCGCCACCGCTGCTGGCTTCTGGAAGGCCACGGGTCGTGATAAAGTCATTTACA 492
Ref_DNA         CCAATCGCGCCACCGCTGCTGGCTTCTGGAAGGCCACGGGTCGTGATAAAGTCATTTACA 524
Ref_mRNA        CCAATCGCGCCACCGCTGCTGGCTTCTGGAAGGCCACGGGTCGTGATAAAGTCATTTACA 457
                ************************************************************

M-yl_cDNA       CCAACTCCCGCCGAATTGGTATGAGAAAGACTCTTGTTTTTTATAAAGGTCGCGCCCCTC 532
WT_yl_cDNA      CCAACTCCCGCCGAATTGGTATGAGAAAGACTCTTGTTTTTTATAAAGGTCGCGCCCCTC 532
WT_sc_cDNA      CCAACTCCCGCCGAATTGGTATGAGAAAGACTCTTGTTTTTTATAAAGGTCGCGCCCCTC 503
M_sc_cDNA       CCAACTCCCGCCGAATTG------------------------------------------ 446
WT_sc_gDNA      CCAACTCCCGCCGAATTGGTATGAGAAAGACTCTTGTTTTTTATAAAGGTCGCGCCCCTC 553
M_sc_gDNA       CCAACTCCCGCCGAATTGGTATGAGAAAGACTCTTGTTTTTTATAAAGGTCGCGCCCCTC 552
Ref_DNA         CCAACTCCCGCCGAATTGGTATGAGAAAGACTCTTGTTTTTTATAAAGGTCGCGCCCCTC 584
Ref_mRNA        CCAACTCCCGCCGAATTGGTATGAGAAAGACTCTTGTTTTTTATAAAGGTCGCGCCCCTC 517
                ******************
                 Exon 2          Mutant seed coat specific alternate splicing
M-yl_cDNA       ACGGCCAGAAGTCTGATTGGATCATGCATGAATATCGCCTCGATGAAATTTCCACCTCCC 592
WT_yl_cDNA      ACGGCCAGAAGTCTGATTGGATCATGCATGAATATCGCCTCGATGAAATTTCCACCTCCC 592
WT_sc_cDNA      ACGGCCAGAAGTCTGATTGGATCATGCATGAATATCGCCTCGATGAAATTTCCACCTCCC 563
M_sc_cDNA       ------------------------------------------------------------ 446
WT_sc_gDNA      ACGGCCAGAAGTCTGATTGGATCATGCATGAATATCGCCTCGATGAAATTTCCACCTCCC 613
M_sc_gDNA       ACGGCCAGAAGTCTGATTGGATCATGCATGAATATCGCCTCGATGAAATTTCCACCTCCC 612
Ref_DNA         ACGGCCAGAAGTCTGATTGGATCATGCATGAATATCGCCTCGATGAAATTTCCACCTCCC 644
Ref_mRNA        ACGGCCAGAAGTCTGATTGGATCATGCATGAATATCGCCTCGATGAAATTTCCACCTCCC 577


M-yl_cDNA       AATCCAGTAA-------------------------------------------------- 602
WT_yl_cDNA      AATCCAGTAA-------------------------------------------------- 602
WT_sc_cDNA      AATCCAGTAA-------------------------------------------------- 573
M_sc_cDNA       ------------------------------------------------------------ 446
WT_sc_gDNA      AATCCAGTAATGTATGTTTGTTTGTCTTATCTCGTTAAGAATTAGAAACATAAGTGGGAT 673
M_sc_gDNA       AATCCAGTAATGTATGTTTGTTTGTCTTATCTCGTTAAGAATTAGAAACATAAGTGGGAT 672
Ref_DNA         AATCCAGTAATGTATGTCTGTTTGTCTTATCTCGTTAAGAATTAGAAACATAAGTGGGAT 704
Ref_mRNA        AATCCAGTAA-------------------------------------------------- 587
                          Start of Intron 2

M-yl_cDNA       ------------------------------------------------------------ 602
WT_yl_cDNA      ------------------------------------------------------------ 602
WT_sc_cDNA      ------------------------------------------------------------ 573
M_sc_cDNA       ------------------------------------------------------------ 446
WT_sc_gDNA      TTTCTTGGATTCCAGTTATGTTTTCCATTCTTAACATGGTATCAGATTAGAGGTTGTTAT 733
M_sc_gDNA       TTTCTTGGATTCCAGTTATGTTTTCCATTCTTAACATGGTATCAGATTAGAGGTTGTTAT 732
Ref_DNA         TTTCTTGGATTCCAGTTCTGTTTTCCATTCTTAACATGGTATCAGATTAGAGGTTGTTAT 764
```

```
Ref_mRNA        ------------------------------------------------------------ 587


M-yl_cDNA       -----------------------------------TATTAAGGCGTCGTCGAGCGGTGTG 627
WT_yl_cDNA      -----------------------------------TATTAAGGCGTCGTCGAGCGGTGTG 627
WT_sc_cDNA      -----------------------------------TATTAAGGCGTCGTCGAGCGGTGTG 598
M_sc_cDNA       ------------------------------------ATTAAGGCGTCGTCGAGCGGTGTG 470
WT_sc_gDNA      TGTTAATGTATCTCTAAATGGTTCGGTTTGATTTAGATTAAGGCGTCGTCGAGCGGTGTG 793
M_sc_gDNA       TGTTAATGTATCTCTAAATGGTTCGGTTTGATTTAGATTAAGGCGTCGTCGAGCGGTGTG 792
Ref_DNA         TGTTAATGTATCTCTAAATGGTTCGGTTTGATTTAGATTAAGGCGTCGTCGAGCGGTGTG 824
Ref_mRNA        -----------------------------------TATTAAGGCGTCGTCGAGCGGTGTG 612
                                                    ************************
                                                   Exon 3 start

M-yl_cDNA       ATTGGAGACGGAGGACAAGAAGAGGGGTGGGTTGTGTGTAGGATATTCAAGAAGAAAAAC 687
WT_yl_cDNA      ATTGGAGACGGAGGACAAGAAGAGGGGTGGGTTGTGTGTAGGATATTCAAGAAGAAAAAC 687
WT_sc_cDNA      ATTGGAGACGGAGGACAAGAAGAGGGGTGGGTTGTGTGTAGGATATTCAAGAAGAAAAAC 658
M_sc_cDNA       ATTGGAGACGGAGGACAAGAAGAGGGGTGGGTTGTGTGTAGGATATTCAAGAAGAAAAAC 530
WT_sc_gDNA      ATTGGAGACGGAGGACAAGAAGAGGGGTGGGTTGTGTGTAGGATATTCAAGAAGAAAAAC 853
M_sc_gDNA       ATTGGAGACGGAGGACAAGAAGAGGGGTGGGTTGTGTGTAGGATATTCAAGAAGAAAAAC 852
Ref_DNA         ATTGGAGACGGAGGACAAGAAGAGGGGTGGGTTGTGTGTAGGATATTCAAGAAGAAAAAC 884
Ref_mRNA        ATTGGAGACGGAGGACAAGAAGAGGGGTGGGTTGTGTGTAGGATATTCAAGAAGAAAAAC 672
                ************************************************************

M-yl_cDNA       CACCACAAAACCCTGGACAGCCCTGTCAGTACTACAATTACCGAAACACCCTCCTCTCTC 747
WT_yl_cDNA      CACCACAAAACCCTGGACAGCCCTGTCAGTACTACAATTACCGAAACACCCTCCTCTCTC 747
WT_sc_cDNA      CACCACAAAACCCTGGACAGCCCTGTCAGTACTACAATTACCGAAACACCCTCCTCTCTC 718
M_sc_cDNA       CACCACAAAACCCTGGACAGCCCTGTCAGTACTACAATTACCGAAACACCCTCCTCTCTC 590
WT_sc_gDNA      CACCACAAAACCCTGGACAGCCCTGTCAGTACTACAATTACCGAAACACCCTCCTCTCTC 913
M_sc_gDNA       CACCACAAAACCCTGGACAGCCCTGTCAGTACTACAATTACCGAAACACCCTCCTCTCTC 912
Ref_DNA         CACCACAAAACCCTGGACAGCCCTGTCAGTACTACAATTACCGAAACACCCTCCTCTCTC 944
Ref_mRNA        CACCACAAAACCCTGGACAGCCCTGTCAGTACTACAATTACCGAAACACCCTCCTCTCTC 732
                ************************************************************

M-yl_cDNA       TTGCTTGATTCTTGCAATGACGGAGGCGTTGGAGCAAATCTTTCATTACATGGGTCGAAG 807
WT_yl_cDNA      TTGCTTGATTCTTGCAATGACGGAGGCGTTGGAGCAAATCTTTCATTACATGGGTCGAAG 807
WT_sc_cDNA      TTGCTTGATTCTTGCAATGACGGAG-CGTTGGAGCAAATCTTTCATTACATGGGTCGAAG 777
M_sc_cDNA       TTGCTTGATTCTTGCAATGACGGAG-CGTTGGAGCAAATCTTTCATTACATGGGTCGAAG 649
WT_sc_gDNA      TTGCTTGATTCTTGCAATGACGGAGGCGTTGGAGCAAATCTTTCATTACATGGGTCGAAG 973
M_sc_gDNA       TTGCTTGATTCTTGCAATGACGGAGGCGTTGGAGCAAATCTTTCATTACATGGGTCGAAG 972
Ref_DNA         TTGCTTGATTCTTGCAATGACGGAG-CGTTGGAGCAAATCTTTCATTACATGGGTCGAAG 1003
Ref_mRNA        TTGCTTGATTCTTGCAATGACGGAG-CGTTGGAGCAAATCTTTCATTACATGGGTCGAAG 791
                ************************* **********************************

M-yl_cDNA       CTGCAAGGACATAGAAGACGGAGACGGAGACGGAGACGGAGACGGCGGCGGTGGGAGATT 867
WT_yl_cDNA      CTGCAAGGACATAGAAGACGGAGACGGAGACGGAGACGGAGACGGCGGCGGTGGGAGATT 867
WT_sc_cDNA      CTGCAAGGACATAGAAGACGGAGACGGAGACGGAGACGGAGACGGCGGCGGTGGGAGATT 837
M_sc_cDNA       CTGCAAGGACATAGAAGACGGAGACGGAGACGGAGACGGAGACGGCGGCGGTGGGAGATT 709
WT_sc_gDNA      CTGCAAGGACATAGAAGACGGAGACGGAGACGGAGACGGAGACGGCGGCGGTGGGAGATT 1033
M_sc_gDNA       CTGCAAGGACATAGAAGACGGAGACGGAGACGGAGACGGAGACGGCGGCGGTGGGAGATT 1032
Ref_DNA         CTGCAAGGACATAGAAGACGGAGACGGAGACGGAGACGGAGACGGCGGCGGTGGGAGATT 1063
Ref_mRNA        CTGCAAGGACATAGAAGACGGAGACGGAGACGGAGACGGAGACGGCGGCGGTGGGAGATT 851
                ************************************************************

M-yl_cDNA       GCTCAGCCCCATCGACACTTCCACTGGCGGTAGTTACCTGGATGGCAGATTCTCCAAACT 927
WT_yl_cDNA      GCTCAGCCCCATCGACACTTCCACTGGCGGTAGTTACCTGGATGGCAGATTCTCCAAACT 927
WT_sc_cDNA      GCTCAGCCCCATCGACACTTCCACTGGCGGTAGTTACCTGGATGGCAGATTCTCCAAACT 897
M_sc_cDNA       GCTCAGCCCCATCGACACTTCCACTGGCGGTAGTTACCTGGATGGCAGATTCTCCAAACT 769
WT_sc_gDNA      GCTCAGCCCCATCGACACTTCCACTGGCGGTAGTTACCTGGATGGCAGATTCTCCAAACT 1093
M_sc_gDNA       GCTCAGCCCCATCGACACTTCCACTGGCGGTAGTTACCTGGATGGCAGATTCTCCAAACT 1092
Ref_DNA         GCTCAGCCCCATCGACACTTCCACTGGCGGTAGTTACCTGGATGGCAGATTCTCCAAACT 1123
Ref_mRNA        GCTCAGCCCCATCGACACTTCCACTGGCGGTAGTTACCTGGATGGCAGATTCTCCAAACT 911
                ************************************************************

M-yl_cDNA       TCCAAACCTCGAAAGCCCCAATTCCACCAGCACCCACAACTGCCACCAACCCATTAACAA 987
WT_yl_cDNA      TCCAAACCTCGAAAGCCCCAATTCCACCAGCACCCACAACTGCCACCAACCCATTAACAA 987
WT_sc_cDNA      TCCAAACCTCGAAAGCCCCAATTCCACCAGCACCCACAACTGCCACCAACCCATTAACAA 957
M_sc_cDNA       TCCAAACCTCGAAAGCCCCAATTCCACCAGCACCCACAACTGCCACCAACCCATTAACAA 829
WT_sc_gDNA      TCCAAACCTCGAAAGCCCCAATTCCACCAGCACCCACAACTGCCACCAACCCATTAACAA 1153
M_sc_gDNA       TCCAAACCTCGAAAGCCCCAATTCCACCAGCACCCACAACTGCCACCAACCCATTAACAA 1152
Ref_DNA         TCCAAACCTCGAAAGCCCCAATTCCACCAGCACCCACAACTGCCACCAACCCATTAACAA 1183
```

```
Ref_mRNA        TCCAAACCTCGAAAGCCCCAATTCCACCAGCACCCACAACTGCCACCAACCCATTAACAA 971
                ************************************************************

M-yl_cDNA       CCATGTGGGCCCCACCGATCCGATTCTAATTTCCGGCTACCAACTGGAGTCCTCTTCTGC 1047
WT_yl_cDNA      CCATGTGGGCCCCACCGATCCGATTCTAATTTCCGGCTACCAACTGGAGTCCTCTTCTGC 1047
WT_sc_cDNA      CCATGTGGGCCCCACCGATCCGATTCTAATTTCCGGCTACCAACTGGAGTCCTCTTCTGC 1017
M_sc_cDNA       CCATGTGGGCCCCACCGATCCGATTCTAATTTCCGGCTACCAACTGGAGTCCTCTTCTGC 889
WT_sc_gDNA      CCATGTGGGCCCCACCGATCCGATTCTAATTTCCGGCTACCAACTGGAGTCCTCTTCTGC 1213
M_sc_gDNA       CCATGTGGGCCCCACCGATCCGATTCTAATTTCCGGCTACCAACTGGAGTCCTCTTCTGC 1212
Ref_DNA         CCATGTGGGCCCCACCGATCCGATTCTAATTTCCGGCTACCAACTGGAGTCCTCTTCTGC 1243
Ref_mRNA        CCATGTGGGCCCCACCGATCCGATTCTAATTTCCGGCTACCAACTGGAGTCCTCTTCTGC 1031
                ************************************************************

M-yl_cDNA       CGCCGCGGCTACAGCTCCTCACAACTGGGCGGCGTTCGACCGGCTGGTGGCCTCGCAACT 1107
WT_yl_cDNA      CGCCGCGGCTACAGCTCCTCACAACTGGGCGGCGTTCGACCGGCTGGTGGCCTCGCAACT 1107
WT_sc_cDNA      CGCCGCGGCTACAGCTCCTCACAACTGGGCGGCGTTCGACCGGCTGGTGGCCTCGCAACT 1077
M_sc_cDNA       CGCCGCGGCTACAGCTCCTCACAACTGGGCGGCGTTCGACCGGCTGGTGGCCTCGCAACT 949
WT_sc_gDNA      CGCCGCGGCTACAGCTCCTCACAACTGGGCGGCGTTCGACCGGCTGGTGGCCTCGCAACT 1273
M_sc_gDNA       CGCCGCGGCTACAGCTCCTCACAACTGGGCGGCGTTCGACCGGCTGGTGGCCTCGCAACT 1272
Ref_DNA         CGCCGCGGCTACAGCTCCTCACAACTGGGCGGCGTTCGACCGGCTGGTGGCCTCGCAACT 1303
Ref_mRNA        CGCCGCGGCTACAGCTCCTCACAACTGGGCGGCGTTCGACCGGCTGGTGGCCTCGCAACT 1091
                ************************************************************

M-yl_cDNA       GAACGGCCAAATTGAAGTGTCCAATATGATTTATTACAATGACCAATTACCAACTCCACC 1167
WT_yl_cDNA      GAACGGCCAAATTGAAGTGTCCAATATGATTTATTACAATGACCAATTACCAACTCCACC 1167
WT_sc_cDNA      GAACGGCCAAATTGAAGTGTCCAATATGATTTATTACAATGACCAATTACCAACTCCACC 1137
M_sc_cDNA       GAACGGCCAAATTGAAGTGTCCAATATGATTTATTACAATGACCAATTACCAACTCCACC 1009
WT_sc_gDNA      GAACGGCCAAATTGAAGTGTCCAATATGATTTATTACAATGACCAATTACCAACTCCACC 1333
M_sc_gDNA       GAACGGCCAAATTGAAGTGTCCAATATGATTTATTACAATGACCAATTACCAACTCCACC 1332
Ref_DNA         GAACGGCCAAATTGAAGTGTCCAATATGATTTATTACAATGACCAATTACCAACTCCACC 1363
Ref_mRNA        GAACGGCCAAATTGAAGTGTCCAATATGATTTATTACAATGACCAATTACCAACTCCACC 1151
                ************************************************************

M-yl_cDNA       AACACTACGCGGAACGACATCGTTTTCGTCCAAATCATCCTCCTCTTCTTACACCGCCGC 1227
WT_yl_cDNA      AACACTACGCGGAACGACATCGTTTTCGTCCAAATCATCCTCCTCTTCTTACACCGCCGC 1227
WT_sc_cDNA      AACACTACGCGGAACGACATCGTTTTCGTCCAAATCATCCTCCTCTTCTTACACCGCCGC 1197
M_sc_cDNA       AACACTACGCGGAACGACATCGTTTTCGTCCAAATCATCCTCCTCTTCTTACACCGCCGC 1069
WT_sc_gDNA      AACACTACGCGGAACGACATCGTTTTCGTCCAAATCATCCTCCTCTTCTTACACCGCCGC 1393
M_sc_gDNA       AACACTACGCGGAACGACATCGTTTTCGTCCAAATCATCCTCCTCTTCTTACACCGCCGC 1392
Ref_DNA         AACACTACGCGGAACGACATCGTTTTCGTCCAAATCATCCTCCTCTTCTTACACCGCCGC 1423
Ref_mRNA        AACACTACGCGGAACGACATCGTTTTCGTCCAAATCATCCTCCTCTTCTTACACCGCCGC 1211
                ************************************************************

M-yl_cDNA       CGCCGGTCAGGATTACAACAACGTGGACACAGAGCTGTGGAGCTTTGCGAAGTTGTCGTC 1287
WT_yl_cDNA      CGCCGGTCAGGATTACAACAACGTGGACACAGAGCTGTGGAGCTTTGCGAAGTTGTCGTC 1287
WT_sc_cDNA      CGCCGGTCAGGATTACAACAACGTGGACACAGAGCTGTGGAGCTTTGCGAAGTTGTCGTC 1257
M_sc_cDNA       CGCCGGTCAGGATTACAACAACGTGGACACAGAGCTGTGGAGCTTTGCGAAGTTGTCGTC 1129
WT_sc_gDNA      CGCCGGTCAGGATTACAACAACGTGGACACAGAGCTGTGGAGCTTTGCGAAGTTGTCGTC 1453
M_sc_gDNA       CGCCGGTCAGGATTACAACAACGTGGACACAGAGCTGTGGAGCTTTGCGAAGTTGTCGTC 1452
Ref_DNA         CGCCGGTCAGGATTACAACAACGTGGACACAGAGCTGTGGAGCTTTGCGAAGTTGTCGTC 1483
Ref_mRNA        CGCCGGTCAGGATTACAACAACGTGGACACAGAGCTGTGGAGCTTTGCGAAGTTGTCGTC 1271
                ************************************************************

M-yl_cDNA       ATCCCTGTCGTCGTCGTCTGACCCATTATGCCACGTGTCCAACACTCCAATATAGCTGCT 1347
WT_yl_cDNA      ATCCCTGTCGTCGTCGTCTGACCCATTATGCCACGTGTCCAACACTCCAATATAGCTGCT 1347
WT_sc_cDNA      ATCCCTGTCGTCGTCGTCTGACCCATTATGCCACGTGTCCAACACTCCAATATAGCTGCT 1317
M_sc_cDNA       ATCCCTGTCGTCGTCGTCTGACCCATTATGCCACGTGTCCAACACTCCAATATAGCTGCT 1189
WT_sc_gDNA      ATCCCTGTCGTCGTCGTCTGACCCATTATGCCACGTGTCCAACACTCCAATATAGCTGCT 1513
M_sc_gDNA       ATCCCTGTCGTCGTCGTCTGACCCATTATGCCACGTGTCCAACACTCCAATATAGCTGCT 1512
Ref_DNA         ATCCCTGTCGTCGTCGTCTGACCCATTATGCCACGTGTCCAACACTCCAATATAGCTGCT 1543
Ref_mRNA        ATCCCTGTCGTCGTCGTCTGACCCATTATGCCACGTGTCCAACACTCCAATATAGCTGCT 1331
                ************************************************************

M-yl_cDNA       ACTGAGAATCATCCACGCGAAGAGAGAGAAAGAGAGAAGAATTTGAAGAACAAGGTT--- 1404
WT_yl_cDNA      ACTGAGAATCATCCACGCGAAGAGAGAGAAAGAGAGAAGAATTTGAAGAACAAGGTT--- 1404
WT_sc_cDNA      ACTGAGAATCATCCACGCCGAAG------------------------------------- 1340
M_sc_cDNA       ACTGAGAATCATCCACGC------------------------------------------ 1207
WT_sc_gDNA      ACTGAGAATCATCCACGCGAAGAGAGAGAAAGAGAGAAGAATTTGAGAAACAAGGTTT-- 1571
M_sc_gDNA       ACTGAGAATCATCCACGCGAAGAGAGAGAAAGAGAGAAGAATTTGAGAAACCAAGGTTT- 1571
Ref_DNA         ACTGAGAATCATCCACGCGAAGAGAGAGAAAGAGAGAAGAATTTGAAGACAAGGTTTTTA 1603
Ref_mRNA        ACTGAGAATCATCCACGCGAAGAGAGAGAAAGAGAGAAGAATTTGAAGACAAGGTTTTTA 1391
```

```
                ******************



M-yl_cDNA       ---------------------------------        1404
WT_yl_cDNA      ---------------------------------        1404
WT_sc_cDNA      ---------------------------------        1340
M_sc_cDNA       ---------------------------------        1207
WT_sc_gDNA      ---------------------------------        1571
M_sc_gDNA       ---------------------------------        1571
Ref_DNA         ATTAATTAATTAAATATTTACGTATGTGATGAA        1636
Ref_mRNA        ATTAATTAATTAAATATTTACGTATGTGATGAA        1424
```
