# Supplementary material for: Alternative Splicing of NAC Transcription Factor Gene CmNST1 Is Associated with Naked Seed Mutation in Pumpkin, Cucurbita moschata
Source: Genes (Basel). 2023 Apr 23;14(5):962. doi: 10.3390/genes14050962 (PMC10217548; doi:10.3390/genes14050962)
Supplement: Supplementary file 1 [file genes-14-00962-s001.zip › Supplemental File 2_final.pdf]

Supplemental file 2. Genomic and mRNA sequences of *CmNST1* in C. maxima (Cmax), C. pepo (Cp) and *C. moschata* and alignment of these sequences. Cp = *C. pepo*, Cma = C. *maxima*, WT = wildtype (HS-A) of *C. moschata*. Cp and Cma sequences are downloaded from public database (https://cucurbitgenomics.org/). Note than annotation of *C. pepo* in the database is incorrect.

A. gDNA and cDNA/mRNA sequences in FASTA format

```
>CMa_gDNA

CACACACACCCAAATGAGTATCTCCGTGAATGGCCACTCCCAAGTCCCTCCCGGCTTCCGCTTCCACCCCACCGAAGAGGAGCTTCTCCACTACTAC
CTCCGCAAGAAGCTCTCCTTCCACACCGTCGATTTGGATGTCATTCCCGACGTCGACCTCAACAAGCTCGAGCCGTGGGACATCCAAGGTACGCATC
TCATTTTCTCCTCTCGTTTTTATTGCCTTTCCTCCTCTCATTCAATTACACTTTCTAGAGAAATGCAAGATCGGAACCACTCCCCAAAACGATTGGT
ACTTTTTTAGTCATAAAGATAAAAAGTATCCCACCGGTACTCGCACCAATCGTGCCACCGCTGCTGGCTTCTGGAAGGCCACTGGTCGTGATAAAGT
CATTTACACTAACTGCCACCGAATTGGTATGCGAAAGACTCTTGTTTTTTACAAGGGTCGCGCCCCTCACGGCCAAAAGTCTGATTGGATCATGCAT
GAATATCGCCTCGATGAAATTTCCACCACCCAATCCAGTAATGTATGTCTCTTTGTCTTATCTCGTTAAGAATTAAAACTCTTGGATCCCACTTATG
TTTTTCATTCATAACACGGTATTAGTTCAAAGCTTCTTATTGTTAATGTATCTCCAAATGGTTCGATTTGACTTAGATTAAGGCGTCGTCGAGCAGT
GTGATTGGAGACGGAGGCCAAGAAGAGGGGTGGGTTGTGTGTAGGATATTCAAGAAGAAAAATCACCACAAAACCCTGGACAGCCCTGTCAGTACTA
CAATTACTGAATCACCCTCCTCTCTCTTGCTTAATTCTTGCAATGACGGAGCCTTGGAGCAAATCTTTCATTACATGGGTCAAACCTGCAAGGACAT
CGAAGACGGAGACGGAGACGGAGACGGCGGCGGCGGTGGGAGATTGCTCAGCCCCATCGACACATCCTCCGGCGGTAGTTACCTGGATGGCAGATTC
TCCAAACTTCCAAACCTCGAAAGCCCCAATTCCACCAGCACCCACAACTGCCACCAACCCATTAACAACCATGTGGGCCCCACCGATCCGATTCTAA
TTCCCGGCTACCAACTTGAGTCCTCTTCTGCCGCCGCCGCCGCTTCAGCTCCTCACAACTGGGCGGCGTTCGACCGGCTGGTGGCCTCGCAACTGAA
CGGCCAAATTGAAGTGTCCAATATGATTTATTACAGTGACCAATTACCAACTCCACCAACACTACGCGGAACGACATCGTTTTCGTCTAAATCATCC
TCCTCTTCTTACACCGCCGCCGCCGGTCAGGATTACAACAACGTGGACACAGAGCTGTGGAGCTTTGCGAAGTTGTCGTCATCCCTGTCGTCGTCAT
CTGACCCATTATGCCACGTGTCCAACACTCCAATATAGCTGCTACTGAGAATCATCCACTCGAAGAGAGAGAAGAATTTGAAGACAAG
>Cma_mRNA

CACACACACCCAAATGAGTATCTCCGTGAATGGCCACTCCCAAGTCCCTCCCGGCTTCCGCTTCCACCCCACCGAAGAGGAGCTTCTCCACTACTAC
CTCCGCAAGAAGCTCTCCTTCCACACCGTCGATTTGGATGTCATTCCCGACGTCGACCTCAACAAGCTCGAGCCGTGGGACATCCAAGAGAAATGCA
AGATCGGAACCACTCCCCAAAACGATTGGTACTTTTTTAGTCATAAAGATAAAAAGTATCCCACCGGTACTCGCACCAATCGTGCCACCGCTGCTGG
CTTCTGGAAGGCCACTGGTCGTGATAAAGTCATTTACACTAACTGCCACCGAATTGGTATGCGAAAGACTCTTGTTTTTTACAAGGGTCGCGCCCCT
CACGGCCAAAAGTCTGATTGGATCATGCATGAATATCGCCTCGATGAAATTTCCACCACCCAATCCAGTAATATTAAGGCGTCGTCGAGCAGTGTGA
TTGGAGACGGAGGCCAAGAAGAGGGGTGGGTTGTGTGTAGGATATTCAAGAAGAAAAATCACCACAAAACCCTGGACAGCCCTGTCAGTACTACAAT
TACTGAATCACCCTCCTCTCTCTTGCTTAATTCTTGCAATGACGGAGCCTTGGAGCAAATCTTTCATTACATGGGTCAAACCTGCAAGGACATCGAA
GACGGAGACGGAGACGGAGACGGCGGCGGCGGTGGGAGATTGCTCAGCCCCATCGACACATCCTCCGGCGGTAGTTACCTGGATGGCAGATTCTCCA
AACTTCCAAACCTCGAAAGCCCCAATTCCACCAGCACCCACAACTGCCACCAACCCATTAACAACCATGTGGGCCCCACCGATCCGATTCTAATTCC
CGGCTACCAACTTGAGTCCTCTTCTGCCGCCGCCGCCGCTTCAGCTCCTCACAACTGGGCGGCGTTCGACCGGCTGGTGGCCTCGCAACTGAACGGC
CAAATTGAAGTGTCCAATATGATTTATTACAGTGACCAATTACCAACTCCACCAACACTACGCGGAACGACATCGTTTTCGTCTAAATCATCCTCCT
CTTCTTACACCGCCGCCGCCGGTCAGGATTACAACAACGTGGACACAGAGCTGTGGAGCTTTGCGAAGTTGTCGTCATCCCTGTCGTCGTCATCTGA
CCCATTATGCCACGTGTCCAACACTCCAATATAGCTGCTACTGAGAATCATCCACTCGAAGAGAGAGAAGAATTTGAAGACAAG

>Cp_gDNA

ATCTCCGTGAATGGCCACTCCCAAGTCCCTCCCGGCTTCCGCTTCCACCCCACCGAAGAGGAGCTCCTCCACTACTACCTCCGCAAGAAGCTCTCCT
TCCACACCGTCGATTTGGATGTCATTCCCGACGTCGATCTCAACAAGCTCGAGCCGTGGGACATCCAAGGTACGTATCTCATTTTCTCCTCTCATTT
TTATTGCCTTTCCTCCTCTCATTCAATTACACTTTCTAGAGAAATGCAAGATCGGAACCACTCCCCAAAACGATTGGTACTTTTTTAGTCATAAAGA
TAAAAAGTATCCCACTGGTACTCGCACCAATCGCGCCACCGCTGCTGGCTTCTGGAAGGCCACTGGTCGTGATAAAGTCATTTACACCAACTCCCGC
CGAATTGGTATGAGAAAGACTCTTGTTTTTTACAAGGGTCGTGCCCCTCATGGCCAGAAGTCTGATTGGATCATGCATGAATATCGCCTCGATGAAA
TTTCCACCTCCCAATCCAGTAATGTATGTTTGTTTGTCTTATCTCGTTAAGAATTAGAAACATAAGTGGGACTTTCTTGGATTCCAGTTATGTTTTT
CATTCTAAACATGGTATCAGATTAGAGGTTGTTATTGTTAATGTATCTCTAAATGGTTCGGTTTGATTTAGATTAAGGCGTCGACGAGCGGTGTGAT
TGGAGACGGAGGACAAGAAGAGGGGTGGGTTGTGTGTAGGATATTCAAGAAGAAAAACCACCACAAAACCCTGGACAGCCCTGTCAGTACTACAATT
ACCGAAACACCCTCCTCTCTCTTGCTTGATTCTTGCAATGACGGAGCGTTGGAGCAAATCTTTCATTACATGGGTCGAAGCTGCAAGGACATAGAAG
ACGGAGACGGCGGCGGCGGCGGTGGGAGATTGCTCAGCCCCATCGACACTTCCACCGGCGGTAGTTACCTGGATGGCAGATTCTCCAAACTTCCAAA
CCTCGAAAGCCCCAATTCCACCAGCACCCACAACTGCCACCAACCCATTAACAACCATGTGGGCCCCACCGATCCGATTCTAATTTCCGGCTACCAA
CTGGAGTCCTCTTCTGCCACCGCCGCCGCGGCTACAGCTCCTCACAACTGGGCGGCGTTCGACCGGCTGGTGGCCTCGCAACTGAACGGCCAAATTG
AAGTGTCCAATATGATTTATTACAATGACCAATTACCAACTCCACCAACACTACGCGGAACGACATCGTTTTCGTCCAAATCATCCTCTTCTTCTTA
CACCGCCGCCGCCGGTCAGGATTACAACAACGTGGACACAGAGCTGTGGAGCTTTGCGAAGTTGTCGTCATCCCTGTCGTCGTCGTCTGACCCATTA
TGCCACGTGTCCAACACTCCAATA
>Cp_mRNA

ATCTCCGTGAATGGCCACTCCCAAGTCCCTCCCGGCTTCCGCTTCCACCCCACCGAAGAGGAGCTCCTCCACTACTACCTCCGCAAGAAGCTCTCCT
TCCACACCGTCGATTTGGATGTCATTCCCGACGTCGATCTCAACAAGCTCGAGCCGTGGGACATCCAAGAGAAATGCAAGATCGGAACCACTCCCCA
AAACGATTGGTACTTTTTTAGTCATAAAGATAAAAAGTATCCCACTGGTACTCGCACCAATCGCGCCACCGCTGCTGGCTTCTGGAAGGCCACTGGT
CGTGATAAAGTCATTTACACCAACTCCCGCCGAATTGGTATGAGAAAGACTCTTGTTTTTTACAAGGGTCGTGCCCCTCATGGCCAGAAGATATTCA
AGAAGAAAAACCACCACAAAACCCTGGACAGCCCTGTCAGTACTACAATTACCGAAACACCCTCCTCTCTCTTGCTTGATTCTTGCAATGACGGAGC
GTTGGAGCAAATCTTTCATTACATGGGTCGAAGCTGCAAGGACATAGAAGACGGAGACGGCGGCGGCGGCGGTGGGAGATTGCTCAGCCCCATCGAC
ACTTCCACCGGCGGTAGTTACCTGGATGGCAGATTCTCCAAACTTCCAAACCTCGAAAGCCCCAATTCCACCAGCACCCACAACTGCCACCAACCCA
TTAACAACCATGTGGGCCCCACCGATCCGATTCTAATTTCCGGCTACCAACTGGAGTCCTCTTCTGCCACCGCCGCCGCGGCTACAGCTCCTCACAA
CTGGGCGGCGTTCGACCGGCTGGTGGCCTCGCAACTGAACGGCCAAATTGAAGTGTCCAATATGATTTATTACAATGACCAATTACCAACTCCACCA
```

ACACTACGCGGAACGACATCGTTTTCGTCCAAATCATCCTCTTCTTCTTACACCGCCGCCGCCGGTCAGGATTACAACAACGTGGACACAGAGCTGT
GGAGCTTTGCGAAGTTGTCGTCATCCCTGTCGTCGTCGTCTGACCCATTATGCCACGTGTCCAACACTCCAATA
>WT_gDNA

GCTCTTCTCCAAGCATCATACTAAAACTATACTTGCTTCAACTATATATGCCAACCCCTCTCTGTTTCCAAACAAAAACATAACACACACACACACA
CACACACACCCAAATGAGTATCTCCGTGAATGGCCACTCCCAAGTCCCTCCCGGCTTCCGCTTCCACCCCACCGAAGAGGAGCTCCTCCACTACTAC
CTCCGCAAGAAGCTCTCCTTCCACACCGTCGATTTGGATGTCATTCCCGATGTCGACCTCAACAAGCTCGAGCCGTGGGACATCCAAGGTACGTATC
TCATTTTCTCCTCTCATTTTTATTGCCTTTCCTCCTCTCATTCAATTACACTTTCTAGAGAAATGCAAGATCGGAACCACTCCTCAAAACGATTGGT
ACTTTTTTAGTCATAAAGATAAAAAGTATCCCACCGGTACTCGCACCAATCGCGCCACCGCTGCTGGCTTCTGGAAGGCCACGGGTCGTGATAAAGT
CATTTACACCAACTCCCGCCGAATTGGTATGAGAAAGACTCTTGTTTTTTATAAAGGTCGCGCCCCTCACGGCCAGAAGTCTGATTGGATCATGCAT
GAATATCGCCTCGATGAAATTTCCACCTCCCAATCCAGTAATGTATGTTTGTTTGTCTTATCTCGTTAAGAATTAGAAACATAAGTGGGATTTTCTT
GGATTCCAGTTATGTTTTCCATTCTTAACATGGTATCAGATTAGAGGTTGTTATTGTTAATGTATCTCTAAATGGTTCGGTTTGATTTAGATTAAGG
CGTCGTCGAGCGGTGTGATTGGAGACGGAGGACAAGAAGAGGGGTGGGTTGTGTGTAGGATATTCAAGAAGAAAAACCACCACAAAACCCTGGACAG
CCCTGTCAGTACTACAATTACCGAAACACCCTCCTCTCTCTTGCTTGATTCTTGCAATGACGGAGGCGTTGGAGCAAATCTTTCATTACATGGGTCG
AAGCTGCAAGGACATAGAAGACGGAGACGGAGACGGAGACGGAGACGGCGGCGGTGGGAGATTGCTCAGCCCCATCGACACTTCCACTGGCGGTAGT
TACCTGGATGGCAGATTCTCCAAACTTCCAAACCTCGAAAGCCCCAATTCCACCAGCACCCACAACTGCCACCAACCCATTAACAACCATGTGGGCC
CCACCGATCCGATTCTAATTTCCGGCTACCAACTGGAGTCCTCTTCTGCCGCCGCGGCTACAGCTCCTCACAACTGGGCGGCGTTCGACCGGCTGGT
GGCCTCGCAACTGAACGGCCAAATTGAAGTGTCCAATATGATTTATTACAATGACCAATTACCAACTCCACCAACACTACGCGGAACGACATCGTTT
TCGTCCAAATCATCCTCCTCTTCTTACACCGCCGCCGCCGGTCAGGATTACAACAACGTGGACACAGAGCTGTGGAGCTTTGCGAAGTTGTCGTCAT
CCCTGTCGTCGTCGTCTGACCCATTATGCCACGTGTCCAACACTCCAATATAGCTGCTACTGAGAATCATCCACGCGAAGAGAGAGAAAGAGAGAAG
AATTTGAGAAACAAGGTTT

>WT_cDNA

TTTTCTCACCCTTCCTTGCTCTTCTCCAAGCATCATACTAAAACTATACTTGCTTCAACTATATATGCCAACCCCTCTCTGTTTCCAAACAAAAACA
TAACACACACACACACACACACACACCCAAATGAGTATCTCCGTGAATGGCCACTCCCAAGTCCCTCCCGGCTTCCGCTTCCACCCCACCGAAGAGG
AGCTCCTCCACTACTACCTCCGCAAGAAGCTCTCCTTCCACACCGTCGATTTGGATGTCATTCCCGATGTCGACCTCAACAAGCTCGAGCCGTGGGA
CATCCAAGAGAAATGCAAGATCGGAACCACTCCTCAAAACGATTGGTACTTTTTTAGTCATAAAGATAAAAAGTATCCCACCGGTACTCGCACCAAT
CGCGCCACCGCTGCTGGCTTCTGGAAGGCCACGGGTCGTGATAAAGTCATTTACACCAACTCCCGCCGAATTGGTATGAGAAAGACTCTTGTTTTTT
ATAAAGGTCGCGCCCCTCACGGCCAGAAGTCTGATTGGATCATGCATGAATATCGCCTCGATGAAATTTCCACCTCCCAATCCAGTAATATTAAGGC
GTCGTCGAGCGGTGTGATTGGAGACGGAGGACAAGAAGAGGGGTGGGTTGTGTGTAGGATATTCAAGAAGAAAAACCACCACAAAACCCTGGACAGC
CCTGTCAGTACTACAATTACCGAAACACCCTCCTCTCTCTTGCTTGATTCTTGCAATGACGGAGCGTTGGAGCAAATCTTTCATTACATGGGTCGAA
GCTGCAAGGACATAGAAGACGGAGACGGAGACGGAGACGGAGACGGCGGCGGTGGGAGATTGCTCAGCCCCATCGACACTTCCACTGGCGGTAGTTA
CCTGGATGGCAGATTCTCCAAACTTCCAAACCTCGAAAGCCCCAATTCCACCAGCACCCACAACTGCCACCAACCCATTAACAACCATGTGGGCCCC
ACCGATCCGATTCTAATTTCCGGCTACCAACTGGAGTCCTCTTCTGCCGCCGCGGCTACAGCTCCTCACAACTGGGCGGCGTTCGACCGGCTGGTGG
CCTCGCAACTGAACGGCCAAATTGAAGTGTCCAATATGATTTATTACAATGACCAATTACCAACTCCACCAACACTACGCGGAACGACATCGTTTTC
GTCCAAATCATCCTCCTCTTCTTACACCGCCGCCGCCGGTCAGGATTACAACAACGTGGACACAGAGCTGTGGAGCTTTGCGAAGTTGTCGTCATCC
CTGTCGTCGTCGTCTGACCCATTATGCCACGTGTCCAACACTCCAATATAGCTGCTACTGAGAATCATCCACGCCGAAG

B. Genomic and mRNA/cDNA alignment among three *Cucurbita* species. Cp = *C. pepo*, Cma = C. *maxima*, WT = wildtype (HS-A) of *C. moschata*. Cp and Cma sequences are downloaded from public database (https://cucurbitgenomics.org/). Note than annotation of *C. pepo* in the database is incorrect.

```
Cp_gDNA      ------------------------------------------------------------  0
Cp_mRNA      ------------------------------------------------------------  0
CMa_gDNA     ------------------------------------------------------------  0
Cma_mRNA     ------------------------------------------------------------  0
WT_gDNA      -----------------GCTCTTCTCCAAGCATCATACTAAAACTATACTTGCTTCAACT  43
WT_cDNA      TTTTCTCACCCTTCCTTGCTCTTCTCCAAGCATCATACTAAAACTATACTTGCTTCAACT  60


Cp_gDNA      ------------------------------------------------------------  0
Cp_mRNA      ------------------------------------------------------------  0
CMa_gDNA     ------------------------------------------------------CACACA  6
Cma_mRNA     ------------------------------------------------------CACACA  6
WT_gDNA      ATATATGCCAACCCCTCTCTGTTTCCAAACAAAAACATAACACACACACACACACACACA  103
WT_cDNA      ATATATGCCAACCCCTCTCTGTTTCCAAACAAAAACATAACACACACACACACACACACA  120
                                                        SSR

Cp_gDNA      -------------ATCTCCGTGAATGGCCACTCCCAAGTCCCTCCCGGCTTCCGCTTCCA  47
Cp_mRNA      -------------ATCTCCGTGAATGGCCACTCCCAAGTCCCTCCCGGCTTCCGCTTCCA  47
CMa_gDNA     CACCCAAATGAGTATCTCCGTGAATGGCCACTCCCAAGTCCCTCCCGGCTTCCGCTTCCA  66
Cma_mRNA     CACCCAAATGAGTATCTCCGTGAATGGCCACTCCCAAGTCCCTCCCGGCTTCCGCTTCCA  66
WT_gDNA      CACCCAAATGAGTATCTCCGTGAATGGCCACTCCCAAGTCCCTCCCGGCTTCCGCTTCCA  163
WT_cDNA      CACCCAAATGAGTATCTCCGTGAATGGCCACTCCCAAGTCCCTCCCGGCTTCCGCTTCCA  180
                          ***********************************************

                    TSS Exon 1
Cp_gDNA      CCCCACCGAAGAGGAGCTCCTCCACTACTACCTCCGCAAGAAGCTCTCCTTCCACACCGT  107
Cp_mRNA      CCCCACCGAAGAGGAGCTCCTCCACTACTACCTCCGCAAGAAGCTCTCCTTCCACACCGT  107
CMa_gDNA     CCCCACCGAAGAGGAGCTTCTCCACTACTACCTCCGCAAGAAGCTCTCCTTCCACACCGT  126
Cma_mRNA     CCCCACCGAAGAGGAGCTTCTCCACTACTACCTCCGCAAGAAGCTCTCCTTCCACACCGT  126
WT_gDNA      CCCCACCGAAGAGGAGCTCCTCCACTACTACCTCCGCAAGAAGCTCTCCTTCCACACCGT  223
WT_cDNA      CCCCACCGAAGAGGAGCTCCTCCACTACTACCTCCGCAAGAAGCTCTCCTTCCACACCGT  240
             ****************** *****************************************

Cp_gDNA      CGATTTGGATGTCATTCCCGACGTCGATCTCAACAAGCTCGAGCCGTGGGACATCCAAGG  167
Cp_mRNA      CGATTTGGATGTCATTCCCGACGTCGATCTCAACAAGCTCGAGCCGTGGGACATCCA---  164
CMa_gDNA     CGATTTGGATGTCATTCCCGACGTCGACCTCAACAAGCTCGAGCCGTGGGACATCCAAGG  186
Cma_mRNA     CGATTTGGATGTCATTCCCGACGTCGACCTCAACAAGCTCGAGCCGTGGGACATCCA---  183
WT_gDNA      CGATTTGGATGTCATTCCCGATGTCGACCTCAACAAGCTCGAGCCGTGGGACATCCAAGG  283
WT_cDNA      CGATTTGGATGTCATTCCCGATGTCGACCTCAACAAGCTCGAGCCGTGGGACATCCA---  297
             ********************* ***** *****************************

                                                        Start of intron 1
Cp_gDNA      TACGTATCTCATTTTCTCCTCTCATTTTTATTGCCTTTCCTCCTCTCATTCAATTACACT  227
Cp_mRNA      ------------------------------------------------------------  164
CMa_gDNA     TACGCATCTCATTTTCTCCTCTCGTTTTTATTGCCTTTCCTCCTCTCATTCAATTACACT  246
Cma_mRNA     ------------------------------------------------------------  183
WT_gDNA      TACGTATCTCATTTTCTCCTCTCATTTTTATTGCCTTTCCTCCTCTCATTCAATTACACT  343
WT_cDNA      ------------------------------------------------------------  297


Cp_gDNA      TTCTAGAGAAATGCAAGATCGGAACCACTCCCCAAAACGATTGGTACTTTTTTAGTCATA  287
Cp_mRNA      ----AGAGAAATGCAAGATCGGAACCACTCCCCAAAACGATTGGTACTTTTTTAGTCATA  220
CMa_gDNA     TTCTAGAGAAATGCAAGATCGGAACCACTCCCCAAAACGATTGGTACTTTTTTAGTCATA  306
Cma_mRNA     ----AGAGAAATGCAAGATCGGAACCACTCCCCAAAACGATTGGTACTTTTTTAGTCATA  239
WT_gDNA      TTCTAGAGAAATGCAAGATCGGAACCACTCCTCAAAACGATTGGTACTTTTTTAGTCATA  403
WT_cDNA      ----AGAGAAATGCAAGATCGGAACCACTCCTCAAAACGATTGGTACTTTTTTAGTCATA  353
                 *************************** ****************************
                  Start of exon 2

Cp_gDNA      AAGATAAAAAGTATCCCACTGGTACTCGCACCAATCGCGCCACCGCTGCTGGCTTCTGGA  347
Cp_mRNA      AAGATAAAAAGTATCCCACTGGTACTCGCACCAATCGCGCCACCGCTGCTGGCTTCTGGA  280
CMa_gDNA     AAGATAAAAAGTATCCCACCGGTACTCGCACCAATCGTGCCACCGCTGCTGGCTTCTGGA  366
Cma_mRNA     AAGATAAAAAGTATCCCACCGGTACTCGCACCAATCGTGCCACCGCTGCTGGCTTCTGGA  299
WT_gDNA      AAGATAAAAAGTATCCCACCGGTACTCGCACCAATCGCGCCACCGCTGCTGGCTTCTGGA  463
WT_cDNA      AAGATAAAAAGTATCCCACCGGTACTCGCACCAATCGCGCCACCGCTGCTGGCTTCTGGA  413
             ******************* ***************** **********************
```

```
Cp_gDNA         AGGCCACTGGTCGTGATAAAGTCATTTACACCAACTCCCGCCGAATTGGTATGAGAAAGA  407
Cp_mRNA         AGGCCACTGGTCGTGATAAAGTCATTTACACCAACTCCCGCCGAATTGGTATGAGAAAGA  340
CMa_gDNA        AGGCCACTGGTCGTGATAAAGTCATTTACACTAACTGCCACCGAATTGGTATGCGAAAGA  426
Cma_mRNA        AGGCCACTGGTCGTGATAAAGTCATTTACACTAACTGCCACCGAATTGGTATGCGAAAGA  359
WT_gDNA         AGGCCACGGGTCGTGATAAAGTCATTTACACCAACTCCCGCCGAATTGGTATGAGAAAGA  523
WT_cDNA         AGGCCACGGGTCGTGATAAAGTCATTTACACCAACTCCCGCCGAATTGGTATGAGAAAGA  473
                ******* *********************** **** ** ************* ******

Cp_gDNA         CTCTTGTTTTTTACAAGGGTCGTGCCCCTCATGGCCAGAAGTCTGATTGGATCATGCATG  467
Cp_mRNA         CTCTTGTTTTTTACAAGGGTCGTGCCCCTCATGGCC------------------------  376
CMa_gDNA        CTCTTGTTTTTTACAAGGGTCGCGCCCCTCACGGCCAAAAGTCTGATTGGATCATGCATG  486
Cma_mRNA        CTCTTGTTTTTTACAAGGGTCGCGCCCCTCACGGCCAAAAGTCTGATTGGATCATGCATG  419
WT_gDNA         CTCTTGTTTTTTATAAAGGTCGCGCCCCTCACGGCCAGAAGTCTGATTGGATCATGCATG  583
WT_cDNA         CTCTTGTTTTTTATAAAGGTCGCGCCCCTCACGGCCAGAAGTCTGATTGGATCATGCATG  533
                ************* ** ***** ******** ****

Cp_gDNA         AATATCGCCTCGATGAAATTTCCACCTCCCAATCCAGTAATGTATGTTTGTTTGTCTTAT  527
Cp_mRNA         ------------------------------------------------------------  376
CMa_gDNA        AATATCGCCTCGATGAAATTTCCACCACCCAATCCAGTAATGTATGTCTCTTTGTCTTAT  546
Cma_mRNA        AATATCGCCTCGATGAAATTTCCACCACCCAATCCAGTAA--------------------  459
WT_gDNA         AATATCGCCTCGATGAAATTTCCACCTCCCAATCCAGTAATGTATGTTTGTTTGTCTTAT  643
WT_cDNA         AATATCGCCTCGATGAAATTTCCACCTCCCAATCCAGTAA--------------------  573
                                                       Start of intron 2

Cp_gDNA         CTCGTTAAGAATTAGAAACATAAGTGGGACTTTCTTGGATTCCAGTTATGTTTTTCATTC  587
Cp_mRNA         ------------------------------------------------------------  376
CMa_gDNA        CTCGTTAAGAATTAAAAC--------------TCTTGGATCCCACTTATGTTTTTCATTC  592
Cma_mRNA        ------------------------------------------------------------  459
WT_gDNA         CTCGTTAAGAATTAGAAACATAAGTGGGATTTTCTTGGATTCCAGTTATGTTTTCCATTC  703
WT_cDNA         ------------------------------------------------------------  573


Cp_gDNA         TAAACATGGTATCAGATTAGAGGTTGTTATTGTTAATGTATCTCTAAATGGTTCGGTTTG  647
Cp_mRNA         ------------------------------------------------------------  376
CMa_gDNA        ATAACACGGTATTAGTTCAAAGCTTCTTATTGTTAATGTATCTCCAAATGGTTCGATTTG  652
Cma_mRNA        ------------------------------------------------------------  459
WT_gDNA         TTAACATGGTATCAGATTAGAGGTTGTTATTGTTAATGTATCTCTAAATGGTTCGGTTTG  763
WT_cDNA         ------------------------------------------------------------  573

Cp_gDNA         ATTTAGATTAAGGCGTCGACGAGCGGTGTGATTGGAGACGGAGGACAAGAAGAGGGGTGG  707
Cp_mRNA         ------------------------------------------------------------  376
CMa_gDNA        ACTTAGATTAAGGCGTCGTCGAGCAGTGTGATTGGAGACGGAGGCCAAGAAGAGGGGTGG  712
Cma_mRNA        -----TATTAAGGCGTCGTCGAGCAGTGTGATTGGAGACGGAGGCCAAGAAGAGGGGTGG  514
WT_gDNA         ATTTAGATTAAGGCGTCGTCGAGCGGTGTGATTGGAGACGGAGGACAAGAAGAGGGGTGG  823
WT_cDNA         -----TATTAAGGCGTCGTCGAGCGGTGTGATTGGAGACGGAGGACAAGAAGAGGGGTGG  628

                     Start of Exon 3
Cp_gDNA         GTTGTGTGTAGGATATTCAAGAAGAAAAACCACCACAAAACCCTGGACAGCCCTGTCAGT  767
Cp_mRNA         -------AGAAGATATTCAAGAAGAAAAACCACCACAAAACCCTGGACAGCCCTGTCAGT  429
CMa_gDNA        GTTGTGTGTAGGATATTCAAGAAGAAAAATCACCACAAAACCCTGGACAGCCCTGTCAGT  772
Cma_mRNA        GTTGTGTGTAGGATATTCAAGAAGAAAAATCACCACAAAACCCTGGACAGCCCTGTCAGT  574
WT_gDNA         GTTGTGTGTAGGATATTCAAGAAGAAAAACCACCACAAAACCCTGGACAGCCCTGTCAGT  883
WT_cDNA         GTTGTGTGTAGGATATTCAAGAAGAAAAACCACCACAAAACCCTGGACAGCCCTGTCAGT  688
                         * ****************** ******************************

Cp_gDNA         ACTACAATTACCGAAACACCCTCCTCTCTCTTGCTTGATTCTTGCAATGACGGAG-CGTT  826
Cp_mRNA         ACTACAATTACCGAAACACCCTCCTCTCTCTTGCTTGATTCTTGCAATGACGGAG-CGTT  488
CMa_gDNA        ACTACAATTACTGAATCACCCTCCTCTCTCTTGCTTAATTCTTGCAATGACGGAG-CCTT  831
Cma_mRNA        ACTACAATTACTGAATCACCCTCCTCTCTCTTGCTTAATTCTTGCAATGACGGAG-CCTT  633
WT_gDNA         ACTACAATTACCGAAACACCCTCCTCTCTCTTGCTTGATTCTTGCAATGACGGAGGCGTT  943
WT_cDNA         ACTACAATTACCGAAACACCCTCCTCTCTCTTGCTTGATTCTTGCAATGACGGAG-CGTT  747
                *********** *** ******************** ****************** * **

Cp_gDNA         GGAGCAAATCTTTCATTACATGGGTCGAAGCTGCAAGGACATAGAAGACGGAGA------  880
Cp_mRNA         GGAGCAAATCTTTCATTACATGGGTCGAAGCTGCAAGGACATAGAAGACGGAGA------  542
CMa_gDNA        GGAGCAAATCTTTCATTACATGGGTCAAACCTGCAAGGACATCGAAGACGGAGACGGAGA  891
Cma_mRNA        GGAGCAAATCTTTCATTACATGGGTCAAACCTGCAAGGACATCGAAGACGGAGACGGAGA  693
WT_gDNA         GGAGCAAATCTTTCATTACATGGGTCGAAGCTGCAAGGACATAGAAGACGGAGACGGAGA  1003
WT_cDNA         GGAGCAAATCTTTCATTACATGGGTCGAAGCTGCAAGGACATAGAAGACGGAGACGGAGA  807
                ************************** ** ************ ***********
```

```
Cp_gDNA       ------CGGCGGCGGCGGCGGTGGGAGATTGCTCAGCCCCATCGACACTTCCACCGGCGG  934
Cp_mRNA       ------CGGCGGCGGCGGCGGTGGGAGATTGCTCAGCCCCATCGACACTTCCACCGGCGG  596
CMa_gDNA      CGGAGACGGC---GGCGGCGGTGGGAGATTGCTCAGCCCCATCGACACATCCTCCGGCGG  948
Cma_mRNA      CGGAGACGGC---GGCGGCGGTGGGAGATTGCTCAGCCCCATCGACACATCCTCCGGCGG  750
WT_gDNA       CGGAGACGGAGACGGCGGCGGTGGGAGATTGCTCAGCCCCATCGACACTTCCACTGGCGG  1063
WT_cDNA       CGGAGACGGAGACGGCGGCGGTGGGAGATTGCTCAGCCCCATCGACACTTCCACTGGCGG  867
                    ***    *********************************** *** * *****

Cp_gDNA       TAGTTACCTGGATGGCAGATTCTCCAAACTTCCAAACCTCGAAAGCCCCAATTCCACCAG  994
Cp_mRNA       TAGTTACCTGGATGGCAGATTCTCCAAACTTCCAAACCTCGAAAGCCCCAATTCCACCAG  656
CMa_gDNA      TAGTTACCTGGATGGCAGATTCTCCAAACTTCCAAACCTCGAAAGCCCCAATTCCACCAG  1008
Cma_mRNA      TAGTTACCTGGATGGCAGATTCTCCAAACTTCCAAACCTCGAAAGCCCCAATTCCACCAG  810
WT_gDNA       TAGTTACCTGGATGGCAGATTCTCCAAACTTCCAAACCTCGAAAGCCCCAATTCCACCAG  1123
WT_cDNA       TAGTTACCTGGATGGCAGATTCTCCAAACTTCCAAACCTCGAAAGCCCCAATTCCACCAG  927
              ************************************************************

Cp_gDNA       CACCCACAACTGCCACCAACCCATTAACAACCATGTGGGCCCCACCGATCCGATTCTAAT  1054
Cp_mRNA       CACCCACAACTGCCACCAACCCATTAACAACCATGTGGGCCCCACCGATCCGATTCTAAT  716
CMa_gDNA      CACCCACAACTGCCACCAACCCATTAACAACCATGTGGGCCCCACCGATCCGATTCTAAT  1068
Cma_mRNA      CACCCACAACTGCCACCAACCCATTAACAACCATGTGGGCCCCACCGATCCGATTCTAAT  870
WT_gDNA       CACCCACAACTGCCACCAACCCATTAACAACCATGTGGGCCCCACCGATCCGATTCTAAT  1183
WT_cDNA       CACCCACAACTGCCACCAACCCATTAACAACCATGTGGGCCCCACCGATCCGATTCTAAT  987
              ************************************************************

Cp_gDNA       TTCCGGCTACCAACTGGAGTCCTCTTCTGCCACCGCCGCCGCGGCTACAGCTCCTCACAA  1114
Cp_mRNA       TTCCGGCTACCAACTGGAGTCCTCTTCTGCCACCGCCGCCGCGGCTACAGCTCCTCACAA  776
CMa_gDNA      TCCCGGCTACCAACTTGAGTCCTCTTCTGCCGCC---GCCGCCGCTTCAGCTCCTCACAA  1125
Cma_mRNA      TCCCGGCTACCAACTTGAGTCCTCTTCTGCCGCC---GCCGCCGCTTCAGCTCCTCACAA  927
WT_gDNA       TTCCGGCTACCAACTGGAGTCCTCTTCTGCCGC------CGCGGCTACAGCTCCTCACAA  1237
WT_cDNA       TTCCGGCTACCAACTGGAGTCCTCTTCTGCCGC------CGCGGCTACAGCTCCTCACAA  1041
              * ************* *************** *      *** *** *************

Cp_gDNA       CTGGGCGGCGTTCGACCGGCTGGTGGCCTCGCAACTGAACGGCCAAATTGAAGTGTCCAA  1174
Cp_mRNA       CTGGGCGGCGTTCGACCGGCTGGTGGCCTCGCAACTGAACGGCCAAATTGAAGTGTCCAA  836
CMa_gDNA      CTGGGCGGCGTTCGACCGGCTGGTGGCCTCGCAACTGAACGGCCAAATTGAAGTGTCCAA  1185
Cma_mRNA      CTGGGCGGCGTTCGACCGGCTGGTGGCCTCGCAACTGAACGGCCAAATTGAAGTGTCCAA  987
WT_gDNA       CTGGGCGGCGTTCGACCGGCTGGTGGCCTCGCAACTGAACGGCCAAATTGAAGTGTCCAA  1297
WT_cDNA       CTGGGCGGCGTTCGACCGGCTGGTGGCCTCGCAACTGAACGGCCAAATTGAAGTGTCCAA  1101
              ************************************************************

Cp_gDNA       TATGATTTATTACAATGACCAATTACCAACTCCACCAACACTACGCGGAACGACATCGTT  1234
Cp_mRNA       TATGATTTATTACAATGACCAATTACCAACTCCACCAACACTACGCGGAACGACATCGTT  896
CMa_gDNA      TATGATTTATTACAGTGACCAATTACCAACTCCACCAACACTACGCGGAACGACATCGTT  1245
Cma_mRNA      TATGATTTATTACAGTGACCAATTACCAACTCCACCAACACTACGCGGAACGACATCGTT  1047
WT_gDNA       TATGATTTATTACAATGACCAATTACCAACTCCACCAACACTACGCGGAACGACATCGTT  1357
WT_cDNA       TATGATTTATTACAATGACCAATTACCAACTCCACCAACACTACGCGGAACGACATCGTT  1161
              ************** *********************************************

Cp_gDNA       TTCGTCCAAATCATCCTCTTCTTCTTACACCGCCGCCGCCGGTCAGGATTACAACAACGT  1294
Cp_mRNA       TTCGTCCAAATCATCCTCTTCTTCTTACACCGCCGCCGCCGGTCAGGATTACAACAACGT  956
CMa_gDNA      TTCGTCTAAATCATCCTCCTCTTCTTACACCGCCGCCGCCGGTCAGGATTACAACAACGT  1305
Cma_mRNA      TTCGTCTAAATCATCCTCCTCTTCTTACACCGCCGCCGCCGGTCAGGATTACAACAACGT  1107
WT_gDNA       TTCGTCCAAATCATCCTCCTCTTCTTACACCGCCGCCGCCGGTCAGGATTACAACAACGT  1417
WT_cDNA       TTCGTCCAAATCATCCTCCTCTTCTTACACCGCCGCCGCCGGTCAGGATTACAACAACGT  1221
              ****** *********** *****************************************

Cp_gDNA       GGACACAGAGCTGTGGAGCTTTGCGAAGTTGTCGTCATCCCTGTCGTCGTCGTCTGACCC  1354
Cp_mRNA       GGACACAGAGCTGTGGAGCTTTGCGAAGTTGTCGTCATCCCTGTCGTCGTCGTCTGACCC  1016
CMa_gDNA      GGACACAGAGCTGTGGAGCTTTGCGAAGTTGTCGTCATCCCTGTCGTCGTCATCTGACCC  1365
Cma_mRNA      GGACACAGAGCTGTGGAGCTTTGCGAAGTTGTCGTCATCCCTGTCGTCGTCATCTGACCC  1167
WT_gDNA       GGACACAGAGCTGTGGAGCTTTGCGAAGTTGTCGTCATCCCTGTCGTCGTCGTCTGACCC  1477
WT_cDNA       GGACACAGAGCTGTGGAGCTTTGCGAAGTTGTCGTCATCCCTGTCGTCGTCGTCTGACCC  1281
              *************************************************** ********

Cp_gDNA       ATTATGCCACGTGTCCAACACTCCAATA--------------------------------  1382
Cp_mRNA       ATTATGCCACGTGTCCAACACTCCAATA--------------------------------  1044
CMa_gDNA      ATTATGCCACGTGTCCAACACTCCAATATAGCTGCTACTGAGAATCATCCACTCGAAGAG  1425
Cma_mRNA      ATTATGCCACGTGTCCAACACTCCAATATAGCTGCTACTGAGAATCATCCACTCGAAGAG  1227
WT_gDNA       ATTATGCCACGTGTCCAACACTCCAATATAGCTGCTACTGAGAATCATCCACGCGAAGAG  1537
WT_cDNA       ATTATGCCACGTGTCCAACACTCCAATATAGCTGCTACTGAGAATCATCCACGCCGAAG-  1340
              ****************************
```

```
Cp_gDNA       ----------------------------------         1382
Cp_mRNA       ----------------------------------         1044
CMa_gDNA      AGAGAAGAATTTGAAGACAAG-------------         1446
Cma_mRNA      AGAGAAGAATTTGAAGACAAG-------------         1248
WT_gDNA       AGAGAAAGAGAGAAGAATTTGAGAAACAAGGTTT         1571
WT_cDNA       ----------------------------------         1340
```
