# Supplementary figures and images for: Alternative Splicing of NAC Transcription Factor Gene CmNST1 Is Associated with Naked Seed Mutation in Pumpkin, Cucurbita moschata
Source: Genes (Basel). 2023 Apr 23;14(5):962. doi: 10.3390/genes14050962 (PMC10217548; doi:10.3390/genes14050962)

## Slide 1
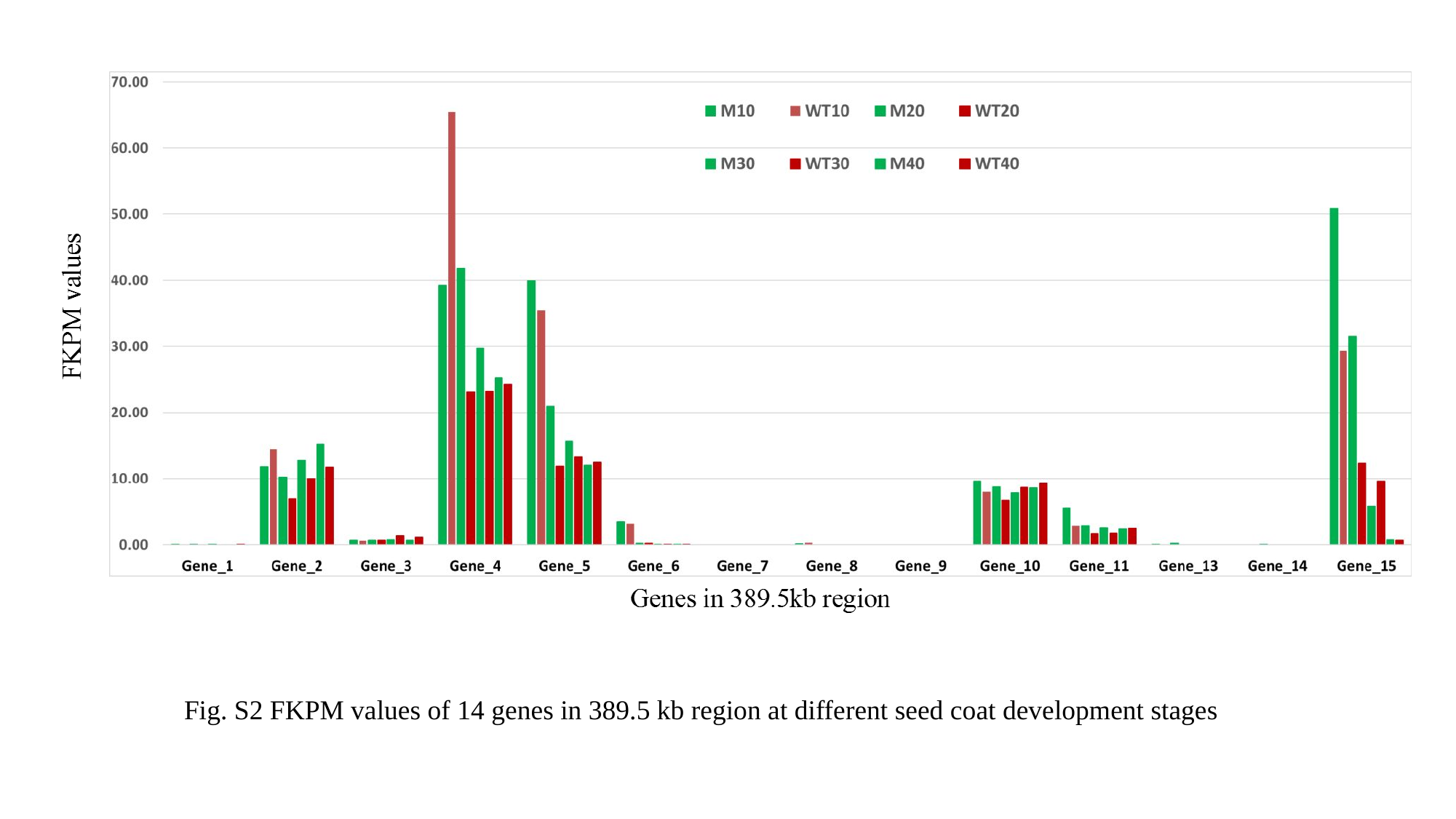

Fig. S2 FKPM values of 14 genes in 389.5 kb region at different seed coat development stages

Supplement: Supplementary file 1 [file genes-14-00962-s001.zip › Fig. S2_v2.0-1.pptx]
